# Photosynthetic rate dominates the seasonal variation of tree intrinsic water-use efficiency in the humid East Asian Monsoon region

**DOI:** 10.1093/treephys/tpag061

**Published:** 2026-04-30

**Authors:** Xinyu He, Yao Li, Ru Huang, Wenling An, Kerstin Treydte, Qingyu Zhao, Junbo Ren, Sainan Liu, Chenxi Xu

**Affiliations:** State Key Laboratory of Lithospheric and Environmental Coevolution, Institute of Geology and Geophysics, Chinese Academy of Sciences, No. 19 Beitucheng Western Road, Chaoyang District, Beijing 100029, China; College of Earth and Planetary Sciences, University of Chinese Academy of Sciences, No. 1 Yanqihu East Ro ad, Huairou District, Beijing 101408, China; State Key Laboratory of Lithospheric and Environmental Coevolution, Institute of Geology and Geophysics, Chinese Academy of Sciences, No. 19 Beitucheng Western Road, Chaoyang District, Beijing 100029, China; College of Earth and Planetary Sciences, University of Chinese Academy of Sciences, No. 1 Yanqihu East Ro ad, Huairou District, Beijing 101408, China; Department of Environment and Biodiversity, Paris-Lodron-University of Salzburg, Hellbrunner Str. 34, Salzburg 5020, Austria; State Key Laboratory of Lithospheric and Environmental Coevolution, Institute of Geology and Geophysics, Chinese Academy of Sciences, No. 19 Beitucheng Western Road, Chaoyang District, Beijing 100029, China; College of Earth and Planetary Sciences, University of Chinese Academy of Sciences, No. 1 Yanqihu East Ro ad, Huairou District, Beijing 101408, China; Research Unit Forest and Soil Ecology, Swiss Federal Institute for Forest, Snow and Landscape Research WSL, Zuercherstrasse 111, Birmensdorf 8903, Switzerland; Oeschger Centre for Climate Change Research, University of Bern, Hochschulstrasse 4, Bern 3012, Switzerland; State Key Laboratory of Lithospheric and Environmental Coevolution, Institute of Geology and Geophysics, Chinese Academy of Sciences, No. 19 Beitucheng Western Road, Chaoyang District, Beijing 100029, China; State Key Laboratory of Lithospheric and Environmental Coevolution, Institute of Geology and Geophysics, Chinese Academy of Sciences, No. 19 Beitucheng Western Road, Chaoyang District, Beijing 100029, China; State Key Laboratory of Lithospheric and Environmental Coevolution, Institute of Geology and Geophysics, Chinese Academy of Sciences, No. 19 Beitucheng Western Road, Chaoyang District, Beijing 100029, China; College of Earth and Planetary Sciences, University of Chinese Academy of Sciences, No. 1 Yanqihu East Ro ad, Huairou District, Beijing 101408, China; State Key Laboratory of Lithospheric and Environmental Coevolution, Institute of Geology and Geophysics, Chinese Academy of Sciences, No. 19 Beitucheng Western Road, Chaoyang District, Beijing 100029, China; College of Earth and Planetary Sciences, University of Chinese Academy of Sciences, No. 1 Yanqihu East Ro ad, Huairou District, Beijing 101408, China

**Keywords:** tree ring, seasonal variation, iWUE, δ^13^C, δ^18^O

## Abstract

Intrinsic water-use efficiency (iWUE) in trees, defined as the ratio of photosynthetic rate (*A*) to stomatal conductance (*g*_s_), is a key indicator characterizing the carbon–water balance in trees. Previous studies have suggested that seasonal variation in iWUE is primarily controlled by *g*_s_ in arid regions; however, the main driving factors in humid regions remain unclear. Therefore, this study utilized 8 years of high-resolution tree-ring δ^13^C and δ^18^O data from *Pinus massoniana* (Lamb.) (coniferous) and *Sassafras tzumu* (Hemsl.) (broadleaf) in the humid East Asian monsoon region to reconstruct the seasonal dynamics of iWUE. We estimated iWUE based on δ^13^C and derived the leaf water δ^18^O enrichment (Δ^18^O_lw_) from δ^18^O to represent *g*_s_, thereby distinguishing the relative contributions of *A* and *g*_s_ to variations in iWUE. Both species exhibited synchronous seasonal iWUE patterns: decreasing from spring to summer before autumn recovery. Dual-isotope analysis revealed that iWUE seasonal variations were primarily driven by fluctuations in *A*, contrasting with *g*_s_-dominated mechanisms in arid regions. Summer iWUE declines resulted from reduced *A*, constrained by relatively low CO_2_ and high temperatures. This study reveals an *A*-dominated regulatory mechanism of iWUE in humid regions, providing a theoretical basis for more accurate predictions of forest carbon–water coupling under varying moisture conditions.

## Introduction

Since the Industrial Revolution, rising atmospheric carbon dioxide concentrations (*C*_a_) have enhanced tree photosynthesis and intrinsic water-use efficiency (iWUE), thereby promoting plant growth, vegetation biomass and soil organic matter (Walker et al. 2021). This process facilitates the fixation of atmospheric carbon dioxide into terrestrial ecosystems, strengthening the role of trees as a carbon sink. Furthermore, if the terrestrial carbon sink continues to intensify, it could reduce the rate of *C*_a_ increase, thus contributing to the mitigation of global climate warming (Mo et al. 2023). As a key physiological parameter, iWUE represents the ratio of the net photosynthetic rate (*A*) to stomatal conductance (*g*_s_) (Farquhar et al. 1982). It reflects the balance between carbon assimilation and transpirational water loss in plants and is fundamental to vegetation–climate interactions and terrestrial water cycles at the global scale (Guerrieri et al. 2019, Li et al. 2023).

Tree-ring stable carbon isotope (δ^13^C) records enable the reconstruction of iWUE over long-term scales, revealing its dynamics from interannual to centennial variations (Huang et al. 2017, Reed et al. 2018, Adams et al. 2020, Treml et al. 2022, Rubio-Cuadrado et al. 2024, Wang et al. 2024). Globally, tree-ring δ^13^C chronologies have consistently shown an increasing trend in iWUE over recent decades (Mathias and Thomas 2021). To disentangle the relative contributions of *A* and *g*_s_ to iWUE trends, a dual-isotope approach combining δ^13^C and stable oxygen isotope ratios (δ^18^O) (Farquhar and Lloyd 1993, Roden et al. 2000, Scheidegger et al. 2000, Farquhar et al. 2007) has been widely employed (Guerrieri et al. 2019, Mathias and Thomas 2021, Lin et al. 2022, Rubio-Cuadrado et al. 2024, Wang et al. 2024). Dual-isotope studies indicate that the rise in iWUE at the global scale is primarily attributed to enhanced *A* driven by increasing *C*_a_ (Guerrieri et al. 2019, Mathias and Thomas 2021). Concurrently, local environmental factors—including temperature (*T*), precipitation (*P*), relative humidity (RH) and vapor pressure deficit (VPD)—also influence iWUE by modulating the balance between *A* and *g*_s_ (McAdam and Brodribb 2015, Dusenge et al. 2019, Grossiord et al. 2020). Specifically, increases in iWUE in humid regions are mainly driven by enhanced *A*, whereas in arid regions they rely more on reduced *g*_s_ (Guerrieri et al. 2019). Furthermore, tree species with different functional traits (e.g., leaf and wood functional types) exhibit distinct iWUE regulatory mechanisms (Mathias and Thomas 2021, Wang et al. 2024). However, recent comprehensive studies have highlighted significant complexities and methodological uncertainties in inferring *g*_s_ from tree-ring δ^18^O (Lin et al. 2022, Carter et al. 2025). These uncertainties stem from interspecific differences in the Péclet effect (Barbour et al. 2021), the potential seasonal variability of the oxygen exchange proportion (*p*_ex_) during cellulose synthesis (Leppä et al. 2026) and the confounding background noise from environmental fluctuations, such as atmospheric water vapor δ^18^O (δ^18^O_v_), temperature and VPD. Therefore, when utilizing the dual-isotope approach, explicitly evaluating these physiological and physical uncertainties is crucial for ensuring the robustness of δ^18^O-derived *g*_s_ interpretations.

However, most existing studies have been conducted at an interannual scale, limiting our understanding of the mechanisms regulating seasonal iWUE dynamics and the response differences among different functional tree types (e.g., conifers vs. broadleaf trees). While interannual studies provide valuable long-term trends, they often mask the physiological responses of trees to rapid environmental fluctuations within a single growing season (Miyahara and Locosselli 2024). Consequently, the emergence of intra-annual stable isotope analysis has become a cornerstone for achieving higher temporal resolution in dendrochronology, enabling researchers to capture tree responses to seasonal climate variations (Wilson and Grinsted 1975, Ogle and McCormac 1994, Vaganov et al. 2009, Schubert and Timmermann 2015, Andreu-Hayles et al. 2022, Kagawa and Battipaglia 2022, Xu et al. 2022, Miyahara and Locosselli 2024, Xu et al. 2024). Recent studies have successfully extended tree-ring δ^13^C applications to seasonal-scale iWUE estimation, with cross-validation through gas exchange measurements and eddy covariance techniques further confirming its reliability (Tang et al. 2023). Investigating the seasonal variation of iWUE is crucial for understanding carbon sequestration, transpiration and related physio-ecological strategies of trees in terrestrial ecosystems, providing important insights into tree growth and carbon–water cycling (Martínez-Sancho et al. 2022, Tang et al. 2023, Portarena et al. 2024).

For example, Martínez-Sancho et al. (2022) found that during extreme summer drought, iWUE increased by 11%, but carbon fixation decreased by 67% due to constraints in cell enlargement and wall thickening, indicating that the increase in iWUE could not fully compensate for drought-induced growth inhibition. Most current research focuses on arid regions, where summer drought typically leads to reduced *g*_s_, thereby elevating iWUE (Barbour et al. 2002, Battipaglia et al. 2014, Martínez-Sancho et al. 2022, Portarena et al. 2024). In contrast, the East Asian monsoon region—a critical global carbon sink—experiences synchronous warm and wet summers with minimal water limitations, potentially leading to a distinctly different iWUE regulatory mechanism compared with arid zones. Nevertheless, the seasonal drivers of iWUE in this region remain poorly understood.

Therefore, this study examined *Pinus massoniana* (Lamb.) (coniferous) and *Sassafras tzumu* (Hemsl.) (broadleaf) in the humid monsoon region of East Asia (sampling site: Lin’an, Zhejiang Province). By collecting tree-ring samples and establishing high-resolution intra-annual isotope sequences, this study aims to physiologically elucidate the seasonal variations in iWUE. First, tree-ring δ^13^C was used to reconstruct the seasonal dynamics of iWUE from 2007 to 2014; second, combined with tree-ring δ^18^O and the oxygen isotope composition of atmospheric precipitation, the degree of leaf water enrichment (Δ^18^O_lw_) was estimated to quantify the contribution of *g*_s_ to changes in iWUE; finally, the relative roles of *A* and *g*_s_ in the seasonal regulation of iWUE were clarified. Specific research objectives include: (i) revealing the seasonal patterns of tree-ring δ^13^C, δ^18^O and iWUE in two functional tree species; (ii) determining the relative contributions of *A* and *g*_s_ to seasonal variations in iWUE; and (iii) identifying key climatic drivers influencing the seasonal changes in iWUE.

## Materials and methods

### Sampling sites and tree species

**Figure 1:**
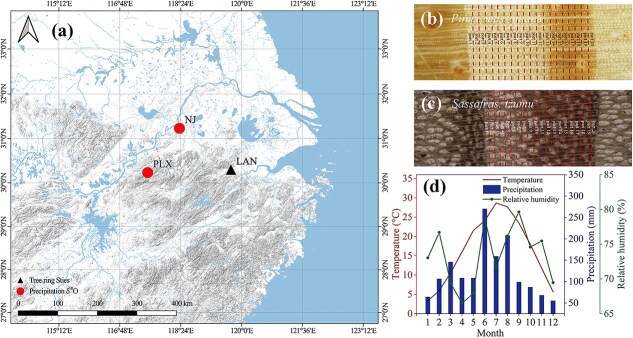
(a) Sampling points and meteorological station locations. Triangles represent the locations of sampling points, Lin’an Meteorological Station and Lin’an Atmospheric Background Station (LAN), while circles indicate the locations of oxygen isotope measurements in atmospheric precipitation; (b, c) Wood anatomical characteristics of *P. massoniana* and *S. tzumu*; (d) Monthly average temperature, precipitation and relative humidity at LAN Station from 2007 to 2014.

Tree-ring samples were collected from a mixed coniferous and broad-leaved forest at the Lin’an Atmospheric Background Station (30.3°N, 119.72°E, 138.6 m a.s.l.) in Hangzhou, Zhejiang Province, southeastern China, using 1-cm-diameter increment borers at a height of 1.3 m above the ground (Figure 1a). *P. massoniana* and *S. tzumu* are the dominant tree species in this forest. *P. massonian*a is a non-porous evergreen coniferous tree (Figure 1b), and *S. tzumu* is a ring-porous deciduous broad-leaved tree (Figure 1c).

### Climate data, atmospheric CO_2_ concentration and precipitation **δ**^18^O

The Lin’an Meteorological Station (30.22°N, 119.70°E, 117.9 m a.s.l.) is ~8 km away from the sampling site (Figure 1a). From 2007 to 2014, the annual mean temperature in this region ranged from 15 to 17 °C, with an average annual precipitation of 1481 mm. The month with the highest precipitation was June, and the primary rainy season occurred between June and August, reflecting typical features of a subtropical monsoon climate (Figure 1d). The mean annual RH was 73.3%, with maximum values observed during summer and autumn.

Vapor pressure deficit (kPa) was calculated based on the monthly temperature and RH data using the formula proposed by Murray (1967):


(1) \begin{equation*} \mathrm{VPD}=0.6112\times \exp \left(\frac{17.08\times T}{T+234.2}\right)\times \left(1-\mathrm{RH}\right) \end{equation*}

where *T* is the monthly average temperature (°C), and RH is the relative humidity (%).

Monthly atmospheric CO_2_ concentration data from 2007 to 2014 were acquired from the Lin’an Atmospheric Background Station. Studies indicate that the atmospheric CO_2_ concentration at this station from 2011 to 2013 exhibits a significant linear relationship with its δ^13^C_CO_2__ value (slope = −0.037, *R*^2^ = 0.8) (Xia et al. 2015, Xia 2016). Based on this, this study utilized the following formula to estimate the δ^13^C_CO_2__ from 2007 to 2014:


(2) \begin{equation*} {\mathrm{\delta}}^{13}{\mathrm{C}}_{\mathrm{CO}_2}=-0.037\mathrm{C}{\mathrm{O}}_2+6.36 \end{equation*}

where δ^13^C_CO_2__ is the carbon isotope value of atmospheric carbon dioxide, CO_2_ is the carbon dioxide concentration and 6.36 is a constant calculated by substituting the CO_2_ and δ^13^C_CO_2__ for 2011.

The precipitation δ^18^O data for 2011 were retrieved from measurements in Anhui (PLX, 30.23°N, 117.53°E) (Duan et al. 2016), while the data for 2012–2014 were from Nanjing (NJ, 32.12°N, 118.95°E) (Li et al. 2020a). Due to the lack of long-term in situ measured data of source water and water vapor isotopes, we utilized the LMDZ4 model data (2007–2010, 2.5° × 3.75° resolution), which has been widely validated and used in tree-ring cellulose δ^18^O research (Hourdin et al. 2006, Shi et al. 2011, An et al. 2019, Huang et al. 2019). Specifically, we extracted both the simulated precipitation δ^18^O (δ^18^O_p_) and the atmospheric water vapor δ^18^O (δ^18^O_v_).

### Stable isotope analysis

At the study site, we collected 40 tree cores from *P. massoniana* and 15 cores from *S. tzumu*. The samples were air-dried, mounted on wooden holders and progressively polished using graded sandpapers until tree-ring boundaries were clearly visible. Ring widths were measured followed by cross-dating to verify chronological accuracy. The period 2007–2014 was chosen for isotopic analysis due to the availability of monthly resolved *C*_a_ and δ^13^C_CO_2__ data (coverage: 2011–2013). High-resolution isotope analysis was conducted on two trees per species (*P. massoniana*: LA02A, LA18A; *S. tzumu*: LA36A, LA39A), which were selected from the cross-dated samples for their nearly parallel ring boundaries. Following separation from wooden holders via hot water soaking, all cores were meticulously cleaned of surface adhesive to eliminate contamination risks. Each annual ring was subjected to serial thin-sectioning using a rotary microtome: initially sectioned at consecutive 60 μm intervals, followed by pooling the sections into 20 parts per annual ring (i.e., each growth year comprises 20 equally divided samples). This high-resolution partitioning approach effectively deconstructs the annual growth process into 20 consecutive temporal units, enabling precise tracking of intra-annual isotopic dynamics during the growing season (refer to Figure 1b and c for schematic illustrations of the ring partitioning in *P. massoniana* and *S. tzumu*). All thin-section samples were subsequently processed for cellulose extraction and isotopic measurements.

Tree-ring α-cellulose was extracted according to a standardized protocol by Xu et al. (2011). Subsequently, 60–100 μg of α-cellulose samples were encapsulated in Sn foil for analyzing the carbon isotope ratios (^13^C/^12^C), while 120–200 μg of α-cellulose samples were encapsulated in silver foil for determining the oxygen isotope ratios (^18^O/^16^O). The carbon and oxygen isotope ratios of tree-ring α-cellulose were measured at the Institute of Geology and Geophysics, Chinese Academy of Sciences, using a coupled isotope ratio mass spectrometer (Delta V Advantage; Thermo Scientific, Germany) and a pyrolysis-based high-temperature conversion elemental analyzer (Flash 2000HT; Thermo Scientific, Germany). The sample stable isotope value were calibrated using a laboratory standard (Merck cellulose) after every set of eight samples. Analytical precision was ±0.23‰ for δ^18^O (*n* = 80) and ±0.05‰ for δ^13^C (*n* = 90) based on repeated measurements of Merck cellulose standards. δ^13^C is the ^13^C/^12^C ratio of the sample normalized to the international standard Vienna Pee Dee Belemnite, while δ^18^O is the ^18^O/^16^O ratio of the sample normalized to Vienna Standard Mean Ocean Water, both expressed in per mil (‰).

### Assigning a date to each section of the tree ring

The *P. massoniana* growing season is from April to October (He et al. 2012). *S. tzumu* flowers in early spring and then grows leaves. The flowering period lasts from mid-February to late March, and the leaves fully develop by mid-to-late April to provide photosynthetic products (Chen et al. 2020). Research indicates that in some deciduous tree species, xylem development initiates before the onset of photosynthesis within the same growing season (Michelot et al. 2012, McCarroll et al. 2017), with non-structural carbohydrates stored during the previous growing season serving as the primary carbon source (Helle and Schleser 2004). Leaf senescence of *S. tzumu* begins in mid-to-late September and continues into early October, coinciding with the onset of dormancy (Jiang et al. 2016). Based on these observations, the growing season of *S. tzumu* is estimated to range from mid-February to mid-to-late September.

**Figure 2:**
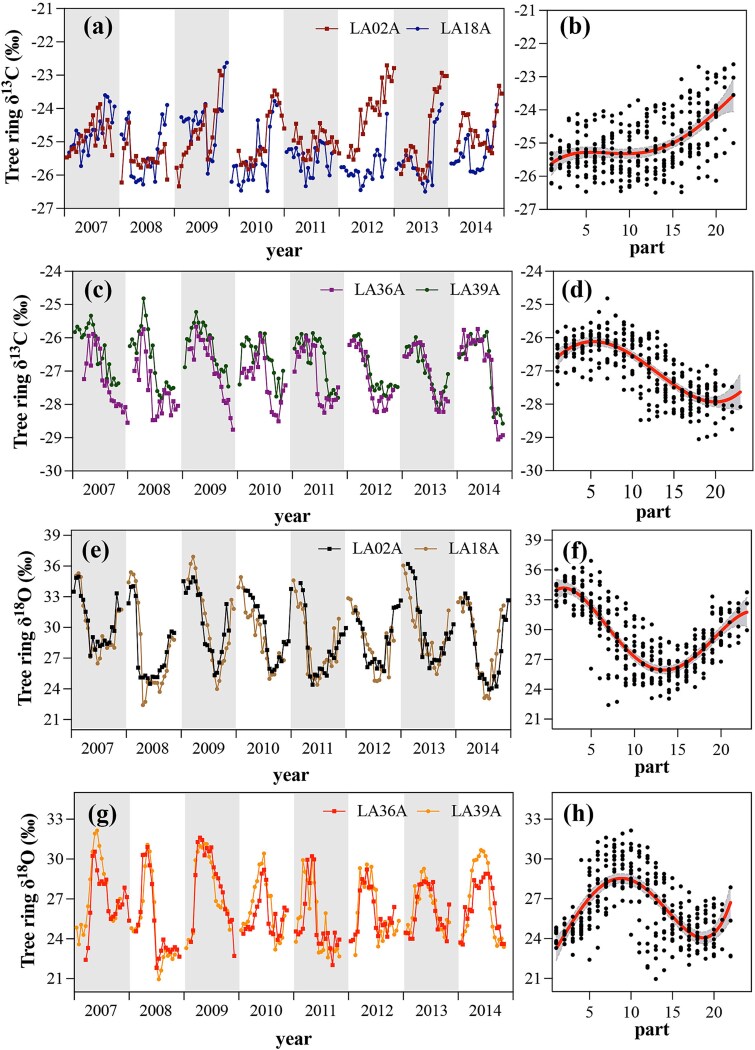
Seasonal variations in synchronized tree-ring δ^13^C (a, c) and their mean seasonal patterns (b, d) for *P. massoniana* (a, b) and *S. tzumu* (c, d) during the period 2007–2014; seasonal variations in synchronized tree-ring δ^18^O (e, g) and their mean seasonal patterns (f, h) for *P. massoniana* (e, f) and *S. tzumu* (g, h) during the period 2007–2014.

To account for inherent individual growth rhythm variations and potential offsets in manual slicing, we first synchronized the intra-annual δ^13^C and δ^18^O curves. By identifying and correcting shifts of one to four segments at the starting positions, we aligned physical segments from different trees to consistent physiological growth stages. Since both isotopes were measured from the same physical slices, a simultaneous shift calibration was applied. The synchronized data are presented in Figure 2, with the original raw measurements provided in Figure S1 available as Supplementary Data at *Tree Physiology* Online.

Near the study area, the absence of systematic observations on the growth patterns of *P. massoniana* and *S. tzumu* using tree radial growth measurement instruments makes it challenging to precisely determine the exact formation periods of each annual ring slice. Although tree-ring growth typically follows an S-shaped function (Ogée et al. 2009, Song et al. 2014), this study focuses on seasonal-scale variation characteristics, with daily-scale errors being within an acceptable range. Based on the phenological traits of *P. massoniana* and *S. tzumu*, we adopted the methodological framework developed by Berkelhammer and Stott (2009). Using the established mathematical formula, we performed the date registration for the subsamples within each annual ring. It is noteworthy that, during the synchronization process to achieve inter-individual phase alignment, the number of effective intra-annual segments for the same species was expanded from the initial 20 to a range of 20–24. Despite variations in *n*_t_ across different years or individuals, the estimation of all dates strictly followed the same mathematical framework to ensure the consistency of the time-allocation criteria. For conciseness, Table S1 available as Supplementary Data at *Tree Physiology* Online only presents the calculated dates for the standard 20-segment scenario, serving as a representative benchmark for this dating methodology.


(3) \begin{equation*} \mathrm{Date}=g_{\mathrm{i}} + g_{\mathrm{l}} (n_{\mathrm{s}} / n_{\mathrm{t}}) \end{equation*}

where *g*_i_ is the start date of growth, *g*_l_ is the length of the growing season, *n*_s_ is the relative position of each section among the total number of sections per tree ring and *n*_t_ is the total number of sections per annual ring. When calculating the corresponding dates for *P. massoniana*, *g*_i_ was set as 1 April, and *g*_l_ was 214 days (from 1 April to 31 October). For *S. tzumu*, *g*_i_ was 15 February, and *g*_l_ was 218 days (from 15 February to 20 September). Based on the calculated dates for each part, the isotopic mean values of tree-ring parts corresponding to each month were obtained.

### Calculation of iWUE

We estimated the iWUE based on high-resolution tree-ring δ^13^C data using two models: the simplified model iWUE_sim_ (Farquhar et al. 1982), which assumes infinite mesophyll conductance (*g*_m_), and the improved model iWUE_mes_ (Ma et al. 2021), which incorporates *g*_m_. The discrimination of atmospheric CO_2_ by plants (Δ^13^C) serves as a critical indicator of plant iWUE, and the formula is presented below:


(4) \begin{equation*} {\Delta}^{13}\mathrm{C}=\frac{\mathrm{\delta}^{13}{\mathrm{C}}_{\mathrm{a}}-{\mathrm{\delta}}^{13}{\mathrm{C}}_{\mathrm{t}}}{1000+{\mathrm{\delta}}^{13}{\mathrm{C}}_{\mathrm{t}}}\times 1000 \end{equation*}

where Δ^13^C is the stable carbon isotope discrimination; δ^13^C_a_ and δ^13^C_t_ are the monthly mean values of δ^13^C for atmospheric CO_2_ and tree-ring cellulose, respectively.


(5) \begin{equation*}\mathrm{iWUE}_{sim}=\frac{A}{g_s}=\frac{C_\mathrm{a}\left(b-{\Delta}^{13}\mathrm{C}\right)}{1.6\left(b-a\right)} \end{equation*}

where *A* is the photosynthetic rate of the plant; *g*_s_ is the stomatal conductance; *C*_a_ is the monthly mean of atmospheric CO_2_ concentration; *a* (4.4‰) is the isotope discrimination during CO_2_ diffusion through stomata; *b* (27‰) is the isotope discrimination value due to the carboxylation; 1.6 is the molar diffusivity ratio of CO_2_ to H_2_O. 


(6) \begin{equation*} {\mathrm{iWUE}}_{\mathrm{mes}} = \frac{{\mathrm{C}}_{\mathrm{a}}}{1.6} \times \frac{b^{\prime } - \Delta - f^{\prime } (\Gamma^{\ast } / C_{\mathrm{a}})}{b^{\prime } - a + \frac{g_{\mathrm{s}}}{g_{\mathrm{m}}} \times (b - a_{\mathrm{m}})} \end{equation*}

(7) \begin{equation*} {\Gamma}^{\ast }=42.7+1.68\left(T-25\right)+0.012{\left(T-25\right)}^2 \end{equation*}

where C_a_ is the monthly mean of atmospheric CO_2_ concentration; *a* (4.4‰) is the isotope discrimination during CO_2_ diffusion through stomata; *b*′ (29‰) and *f*′ (11‰) are the discriminations due to carboxylation and photorespiration, respectively; *a*_m_ (1.8‰) is the discrimination associated with CO_2_ dissolution and diffusion in the mesophyll. The *g*_s_/*g*_m_ ratio is a constant value of 0.79, which was determined through measurements conducted across a broad spectrum of plant species (Ma et al. 2021); Γ^*^ is the CO_2_ compensation point in the absence of mitochondrial respiration (Brooks and Farquhar 1985) and *T* is the average monthly temperature.

### Calculation of the enrichment of leaf water ^18^O over source water (Δ^18^O_lw_)

We calculated the enrichment of ^18^O in tree rings relative to source water (Δ^18^O_t_) following the method of Cheesman and Cernusak (2017):


(8) \begin{equation*} {\Delta}^{18}{\mathrm{O}}_{\mathrm{t}}=\frac{\mathrm{\delta}^{18}{\mathrm{O}}_{\mathrm{t}}-{\mathrm{\delta}}^{18}{\mathrm{O}}_{\mathrm{p}}}{1+\left({\mathrm{\delta}}^{18}{\mathrm{O}}_{\mathrm{p}}/1000\right)} \end{equation*}

where δ^18^O_t_ is the monthly mean δ^18^O value of tree rings, while δ^18^O_p_ is the monthly mean δ^18^O value of precipitation during tree-ring formation. We assume that δ^18^O_p_ is indicative of the oxygen isotope composition of soil water, which serves as source water. Based on the method proposed by Guerrieri et al. (2019), we quantified Δ^18^O_lw_ using the following equation:


(9) \begin{equation*} {\Delta}^{18}{\mathrm{O}}_{\mathrm{lw}}=\frac{\Delta^{18}{\mathrm{O}}_{\mathrm{t}}-\varepsilon wc}{1-p_{\mathrm{x}}p_{\mathrm{ex}}} \end{equation*}

where *p_x_* (~1) represents the proportion of oxygen exchanged with local xylem water during cellulose synthesis, and *p*_ex_ denotes the fraction of oxygen atoms participating in this exchange. Traditionally, *p*_ex_ is assumed to be a constant value of 0.4 (Guerrieri et al. 2019). However, to account for the potential seasonal variability of *p*_ex_ as suggested by recent modeling observations, we additionally conducted a sensitivity simulation. By comparing the standard constant *p*_ex_ scenario (0.4) with a linearly decreasing *p*_ex_ scenario—simulating a dynamic decline of *p*_ex_ from 0.5 to 0.2 across the growing season (Leppä et al. 2026)—we quantified the potential interference of this physiological uncertainty on the derived Δ^18^O_lw_ signal. *εwc* is the temperature-dependent discrimination related to the exchange of carbonyl oxygen atoms with water during cellulose synthesis, *εwc* = 0.0084 *T*^2^ – 0.51 *T* + 33.172 (Sternberg and Ellsworth 2011), *T* is the average monthly temperature.

### Statistical analysis

Pearson and partial correlation analyses were conducted to evaluate the linkage between intra-annual iWUE and Δ^18^O_lw_, and associations of iWUE with key climatic variables (*T*, *P*, RH, VPD and *C*_a_). Partial correlation analysis controlled for covarying effects among environmental factors. Furthermore, Pearson correlation analysis was employed to evaluate the relationships between the simulated Δ^18^O_lw_ under different *p*_ex_ scenarios and iWUE_mes_, aiming to assess the potential impact of physiological uncertainties. The same correlation method was also applied to validate the consistency between the LMDZ4-simulated δ^18^O_v_ and precipitation δ^18^O_p_.

To decouple the potential influence of mathematical coupling between iWUE and *C*_a_—arising from *C*_a_ being an explicit multiplier in the iWUE calculation formula—a ‘null-hypothesis model’ was introduced to isolate true biological signals. We calculated a theoretical slope (*k*_null_), representing the iWUE increase rate assuming a constant carbon isotope discrimination (Δ^13^C). This baseline was compared against the observed slope (*k*_obs_), derived from the linear regression of iWUE against *C*_a_. *A k*_obs_ significantly exceeding *k*_null_ indicates an active biological signal where physiological regulation enhances the iWUE response beyond the mathematical expectation of rising *C*_a_.

To quantify the relative contributions of environmental factors to seasonal variations in iWUE, we applied multiple linear regression analysis.

First, all variables were standardized using Z-score normalization to eliminate scale effects and improve comparability across the model. The standardization formula is:


(10) \begin{equation*} z=\frac{x-\mu }{\mathrm{\sigma}} \end{equation*}

where *x* is the original variable value, *μ* is the mean and *σ* is the standard deviation.

The multiple linear regression analysis requires a linear relationship between the independent and dependent variables. By examining scatter plots and calculating correlation coefficients, we excluded two climatic variables, *P* and RH.

Subsequently, a multiple linear regression model was constructed:


(11) \begin{equation*} \mathrm{iWUE}={\beta}_0+{\beta}_1T+{\beta}_2 \mathrm{VPD}+{\beta}_3{C}_\mathrm{a}+\varepsilon \end{equation*}

where iWUE is the dependent variable, *β*_0_ is the constant (intercept) term, *T*, VPD and *C_a_* are the independent variables, *β*_1_–*β*_3_ are the partial regression coefficients for *T*, *VPD* and *C*_a_, respectively, and *ε* is the residual term.

To check for multicollinearity among the independent variables and accurately quantify the independent explanatory contributions of each environmental factor to the variation in iWUE, we computed the variance inflation factor (VIF), which measures the extent of variance inflation in coefficient estimates due to multicollinearity. A VIF > 10 (or more strictly, >5) indicates severe multicollinearity (O’Brien 2007).

## Results

### Seasonal variations of tree-ring **δ**^13^C and **δ**^18^O

δ^13^C analysis of two specimens per species (*P. massoniana*: LA02A, LA18A; *S. tzumu*: LA36A, LA39A) confirmed synchronous intra-specific variation (Figure 2a and c). After applying the synchronization process to align physiological growth stages, statistical analysis revealed a significant positive correlation between the two *P. massoniana* individuals (*n* = 144, *r* = 0.43, *p* < 0.01), while an even stronger coordinated variation was observed between the two *S. tzumu* individuals (*n* = 149, *r* = 0.78, *p* < 0.01). *Pinus massoniana* exhibited an intra-annual pattern characterized by a shift from ^13^C depletion in the early growing season to pronounced enrichment in the mid-to-late growing season (Figure 2b), though this pattern was not consistently observed across all investigated years. In contrast, *S. tzumu* maintained strict interannual synchrony in δ^13^C variation, displaying a typical triphasic seasonal pattern: initial isotopic enrichment, mid-season depletion and a transient resurgence in the late season (Figure 2d). The seasonal amplitude of δ^13^C variation was similar between the two species, averaging 2.3‰ for *P. massoniana* and 2.4‰ for *S. tzumu*.

Also, both *P. massoniana* and *S. tzumu* exhibited significant intra-specific synchronicity in δ^18^O values following synchronization (Figure 2e and g). Statistical analysis revealed significant positive correlations between the two individuals of *P. massoniana* (*n* = 144, *r* = 0.85, *p* < 0.01) and similarly between the two individuals of *S. tzumu* (*n* = 149, *r* = 0.84, *p* < 0.01). *Pinus massoniana* exhibited strict temporal coherence in interannual δ^18^O seasonal patterns, characterized by a V-shaped trajectory: an initial depletion phase followed by progressive enrichment (Figure 2f). *Sassafras tzumu* displayed a three-phase δ^18^O pattern: early-growing-season enrichment, mid-season depletion and transient late-season resurgence (Figure 2h). The seasonal amplitude of δ^18^O variation was more species-specific compared with δ^13^C, with 9.7‰ for *P. massoniana* and 7.3‰ for *S. tzumu*.

### Seasonal variation of iWUE and Δ^18^O_lw_

The iWUE_sim_ model overestimated δ^13^C-derived iWUE in *P. massoniana* by 50.31 μmol CO_2_ mol^−1^ H_2_O and in *S. tzumu* by 38.13 μmol CO_2_ mol^−1^ H_2_O compared with iWUE_mes_ measurements. Although neglecting *g*_m_ limitations in the iWUE_sim_ approach resulted in systematic overestimation of absolute iWUE values, both models captured seasonal water-use efficiency patterns consistently (Figure S2 available as Supplementary Data at *Tree Physiology* Online). The iWUE_mes_ framework, which explicitly incorporates *g*_m_ constraints, demonstrated superior estimation accuracy. We therefore employed iWUE_mes_-derived values for subsequent physiological analyses.

**Figure 3:**
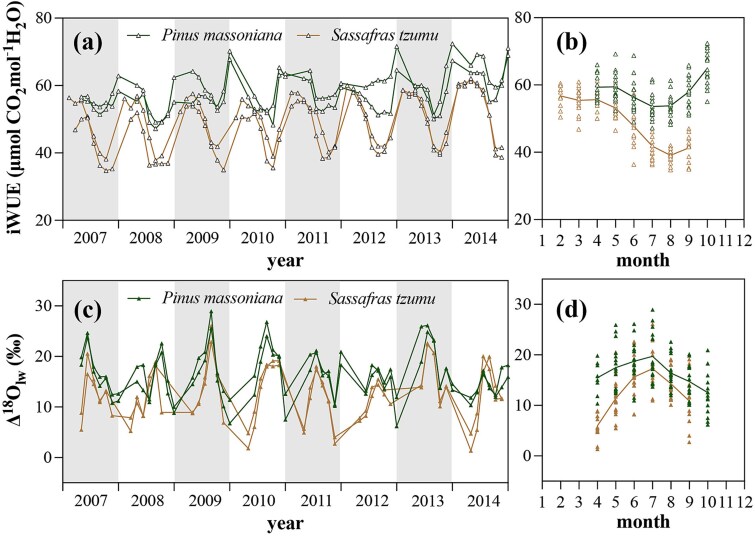
Seasonal variations of iWUE of *P. massoniana* and *S. tzumu* from 2007 to 2014 (a) and their mean seasonal patterns (b); seasonal variations of Δ^18^O_lw_ of *P. massoniana* and *S. tzumu* from 2007 to 2014 (c) and their mean seasonal patterns (d).

Both *P. massoniana* and *S. tzumu* showed significant inter-individual correlations in iWUE (Figure 3a), with correlation coefficients of *r* = 0.73 (*n* = 53, *p* < 0.001) and *r* = 0.95 (*n* = 59, *p* < 0.001), respectively. The two species exhibited synchronized seasonal iWUE patterns during their shared growing seasons, characterized by a decline during the early growth phase, a minimum value in summer and a rebound in autumn (Figure 3b). The mean seasonal amplitudes of iWUE variations were 12.95 μmol CO_2_ mol^−1^ H_2_O for *P. massoniana* and 18.11 μmol CO_2_ mol^−1^ H_2_O for *S. tzumu*. Notably, while *P. massoniana* displayed higher iWUE values overall, its seasonal fluctuation amplitude was smaller compared with *S. tzumu* (Figure 3b).

Both *P. massoniana* and *S. tzumu* exhibited significant inter-individual correlations in Δ^18^O_lw_ (Figure 3c), with correlation coefficients of *r* = 0.87 (*n* = 52, *p* < 0.001) and *r* = 0.95 (*n* = 43, *p* < 0.001), respectively. The two species displayed synchronized seasonal Δ^18^O_lw_ patterns during their shared growing seasons, characterized by an initial rise during the early growth phase, peak values in summer and a subsequent decline in autumn (Figure 3d). The mean seasonal amplitudes of Δ^18^O_lw_ variations were 12‰ for *P. massoniana* and 13.25‰ for *S. tzumu*. Notably, while *P. massoniana* showed higher Δ^18^O_lw_ values overall, its seasonal fluctuation amplitude was smaller compared with *S. tzumu* (Figure 3d).

### Relationships between iWUE, Δ^18^O_lw_ and climatic factors

To discern whether seasonal variations in iWUE were primarily driven by *A* or *g*_s_, we examined the relationship between iWUE and Δ^18^O_lw_. From 2007 to 2014, a significant negative correlation was observed between iWUE and Δ^18^O_lw_ in *P. massoniana* from April to October (*r* = −0.32, *n* = 52, *p* < 0.01) and in *S. tzumu* from April to September (*r* = −0.38, *n* = 43, *p* < 0.01).

**Figure 4:**
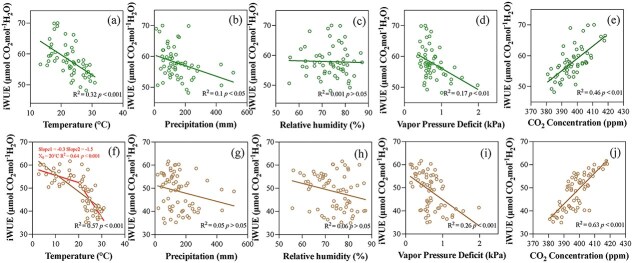
Relationships between iWUE and monthly mean temperature (a, f), precipitation (b, g), relative humidity (c, h), vapor pressure deficit (d, i) and atmospheric CO_2_ concentration (e, j) in *P. massoniana* and *S. tzumu*. The distinct biphasic response of *S. tzumu* iWUE to temperature is highlighted in (f), where the regression lines represent the piecewise linear regressions below and above the 20 °C threshold, with the corresponding slopes indicated by the adjacent numbers.

We further investigated the relationships between iWUE and climate factors. Both *P. massoniana* and *S. tzumu* exhibited similar response patterns of iWUE to climatic factors including *T*, *P*, RH and VPD. Pearson correlation analysis (Figure 4; Table S2 available as Supplementary Data at *Tree Physiology* Online) revealed significantly negative correlations between iWUE and *T* for both *P. massoniana* and *S. tzumu*. Significantly negative correlations were also found between iWUE and VPD in *P. massoniana* and *S. tzumu*. iWUE showed weak correlations with *P* and RH in both species.

To eliminate potential confounding effects from other climatic variables, partial correlation analyses were conducted. The results indicated that significant partial correlations between iWUE and *T* were maintained in *P. massoniana* (Table 1) and *S. tzumu* (Table 2) after controlling for other climatic factors. Notably, no significant correlation was observed between iWUE and VPD when *T* was held constant (Tables 1 and 2).

**Table 1:** Partial correlation coefficients between iWUE and climatic factors for *P. massoniana* (*n* = 56) from 2007 to 2014

	Control variable: *T*	Control variable: *P*	Control variable: RH	Control variable: VPD	Control variable: *C* _a_
*T*		−0.522^***^	−0.574^***^	−0.441^***^	−0.422^***^
*P*	−0.207		0.342^*^	−0.398^**^	−0.339^*^
RH	0.097	0.095		−0.397^**^	0.054
VPD	−0.085	−0.459^***^	−0.545^***^		−0.348^**^
*C*_a_	0.587^***^	0.681^***^	0.679^***^	0.657^***^	

Significance levels: ^***^p < 0.001, ^**^p < 0.01, ^*^p < 0.05.

**Table 2:** Partial correlation coefficients between iWUE and climatic factors for *S. tzumu* (*n* = 64) from 2007 to 2014

	Control variable: *T*	Control variable: *P*	Control variable: RH	Control variable: VPD	Control variable: *C* _a_
*T*		−0.743^***^	−0.761^***^	−0.660^***^	−0.662^***^
*P*	−0.066		−0.137	−0.278^*^	−0.174
RH	−0.283^*^	−0.189		−0.664^***^	−0.398^***^
VPD	−0.151	−0.531^***^	−0.746^***^		−0.341^**^
*C*_a_	0.718^***^	0.789^***^	0.817^***^	0.748^***^	

Significance levels: ^***^p < 0.001, ^**^p < 0.01, ^*^p < 0.05.

### Relationships between iWUE and *C*_a_

Pearson and partial correlation analyses revealed that iWUE for both species was significantly and positively correlated with *C*_a_ (Tables 1 and 2; Table S1 available as Supplementary Data at *Tree Physiology* Online). However, to move beyond the potential artifacts of mathematical background forcing and isolate genuine physiological signals (as detailed in Statistical analysis), we compared the observed sensitivity (*k*_obs_) of iWUE to *C*_a_ against the theoretical null-hypothesis slope (*k*_null_).

The results (Figure S3 available as Supplementary Data at *Tree Physiology* Online) showed that *k*_obs_ substantially exceeded the corresponding *k*_null_ for both species (0.41 vs 0.21 for *P. massoniana*; 0.67 vs 0.23 for *S. tzumu*). This divergence confirms that the observed iWUE increase is not merely a product of the mathematical structure of the calculation formula, but is driven by active biological signals. Specifically, the *k*_obs_ for *S. tzumu* was nearly three times its theoretical value, indicating a significantly more robust active physiological regulation capability in this broadleaf species compared with the needle-leaved *P. massoniana*.

### Relative contributions of environmental factors to the seasonal variation in iWUE

To quantify the relative contributions of environmental factors to the seasonal variation in iWUE, we performed multiple linear regression analyses. In the multiple linear regression model, a larger partial regression coefficient (Beta value) for an environmental factor indicates a stronger independent explanatory power of that factor regarding the variation in iWUE. Since multiple linear regression requires a linear relationship between independent and dependent variables, we excluded *P* and RH due to their weak correlations with iWUE, retaining only *C*_a_, *T* and VPD. Given the correlation between VPD and *T* (*r* = 0.76, *n* = 72, *p* < 0.01), we assessed potential multicollinearity among the predictors by calculating the VIF, which measures the degree to which the variance of a coefficient estimator is increased due to multicollinearity.

**Figure 5:**
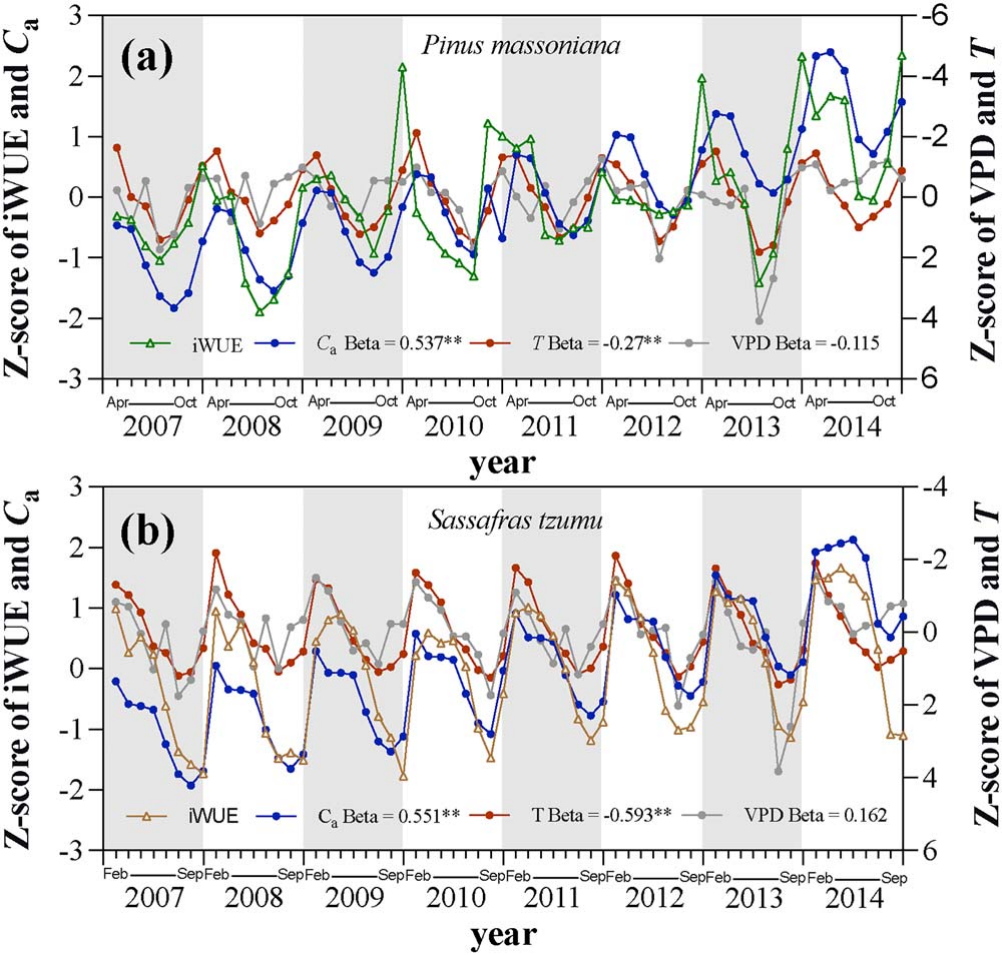
Multiple linear regression analysis of intrinsic water-use efficiency (iWUE) in response to climatic drivers in (a) *P. massoniana* and (b) *S. tzumu*. The numerical values represent standardized coefficients (Beta) for atmospheric CO_2_ concentration (*C*_a_), temperature (*T*) and vapor pressure deficit (VPD), with larger absolute values indicating greater contribution to the variation in iWUE. Significance levels are indicated as: ^**^p < 0.01.

The multiple linear regression model for *P. massoniana* was formulated as (Table S3 available as Supplementary Data at *Tree Physiology* Online): iWUE = 0.537 × *C*_a_ – 0.115 × VPD – 0.27 × *T*. For *S. tzumu*, the regression model was (Table S4 available as Supplementary Data at *Tree Physiology* Online): iWUE = 0.551 × *C*_a_ – 0.593 × *T* + 0.162 × VPD. Multiple linear regression models revealed that *C*_a_, *T* and VPD collectively accounted for a substantial portion of the seasonal variation in iWUE, with a markedly higher explanatory power for *S. tzumu* (*R*^2^ = 0.803) than for *P. massoniana* (*R*^2^ = 0.565; Tables S3 and S4 available as Supplementary Data at *Tree Physiology* Online). *A* consistent driver pattern emerged across both species: *C*_a_ and *T* served as the primary predictors, exerting significant positive and negative effects on iWUE, respectively (Figure 5). Notably, while rising *T* significantly inhibited iWUE, VPD showed no significant independent influence in either model once other factors were accounted for. All models were statistically robust with no substantial multicollinearity (all VIF < 5).

## Discussion

### Seasonal dynamics and interspecific differences in iWUE between *P. massoniana* and *S. tzumu*

During the shared growing season of *P. massoniana* and *S. tzumu*, although the iWUE differed in absolute values between the two species, they exhibited similar seasonal trends: a gradual decrease from spring to summer, followed by an increase in autumn (Figure 3a and b). These findings are consistent with previous studies reporting that although absolute iWUE values vary across tree species, their seasonal patterns tend to be similar (Klein et al. 2013, Morais et al. 2021, Portarena et al. 2024). Moreover, the two species showed comparable responses to climatic variables (Figure 4), suggesting that functional type differences did not alter the consistency in the direction of their responses to environmental drivers.

In terms of interspecific variation, *S. tzumu* exhibited greater seasonal amplitude in iWUE (Figure 3a and b), indicating higher plasticity in water-use regulation. In contrast, *P. massoniana* demonstrated a more conservative water-use strategy, with a narrower range of iWUE variation (Figure 3a and b). Regarding absolute differences, *P. massoniana* consistently exhibited higher iWUE. We attribute this to the generally lower hydraulic conductivity of conifers (non-porous wood) compared with broadleaf trees (ring-porous wood). This anatomical characteristic may enhance their ability to resist embolism under drought conditions (Choat et al. 2012, Carnicer et al. 2013, Guerrieri et al. 2019).

### Photosynthesis-dominated mechanisms control seasonal iWUE dynamics in a humid monsoon region

Inferring *g*_s_ from tree-ring δ^18^O requires accounting for source water influences (Scheidegger et al. 2000). This issue was effectively addressed by calculating leaf water δ^18^O enrichment (Δ^18^O_lw_) using well-established protocols (Barbour et al. 2004, Xu et al. 2018, Guerrieri et al. 2019, Mathias and Thomas 2021).

It should be noted that *S. tzumu* flowers from February to March, before leaf development (Chen et al. 2020). This aligns with tree physiological studies, indicating that deciduous broad-leaved species rely on stored non-structural carbohydrates in the early growth stage (Barbour et al. 2002, Helle and Schleser 2004, Kagawa et al. 2006, Fichtler et al. 2010, Simard et al. 2013). Therefore, Δ^18^O_lw_ was not calculated for *S. tzumu* during these months. We found a significant negative correlation between iWUE and Δ^18^O_lw_ (Figure 3), indicating that iWUE increases with rising *g*_s_. This contradicts the classical inverse iWUE–*g*_s_ theory (Bögelein et al. 2012), confirming that *g*_s_ does not explain iWUE dynamics and establishing *A* as the dominant regulatory factor. This mechanism differs from that observed in summer-dry regions, where trees enhance iWUE through reduced *g*_s_ (Barbour et al. 2002, Battipaglia et al. 2014, Martínez-Sancho et al. 2022, Portarena et al. 2024). Our conclusion is further supported by Ren et al. (2024), who found that variations in bulk-leaf δ^13^C of *Robinia pseudoacacia* in a subtropical humid monsoon climate in China were controlled by photosynthetic capacity (as indicated by leaf nitrogen content), rather than by *g*_s_ regulation.

### Evaluation of uncertainties in inferring stomatal conductance from tree-ring **δ**^18^O

The dual-isotope approach provides a classic theoretical framework for disentangling the forest carbon–water balance (Scheidegger et al. 2000). However, several recent comprehensive studies have highlighted that the linkage between tree-ring δ^18^O and *g*_s_ is indirect and weak, and its inferential process is highly susceptible to interference from various physical and physiological variables (Lin et al. 2022, Carter et al. 2025).

The validity of the dual-isotope model relies on the assumed universality of the Péclet effect and the stability of the effective path length (*L*) (Lin et al. 2022). Addressing this limitation, the two tree species investigated in this study still largely satisfy the prerequisites for applying the inverse Δ^18^O_lw_–*g*_s_ relationship under their specific habitat conditions. On one hand, the Péclet effect in gymnosperms (*P. massoniana*) is typically minimal or negligible (Barbour et al. 2021). Although the absence of this effect diminishes the sensitivity of Δ^18^O_lw_ to *g*_s_, it does not alter their inverse relationship. On the other hand, for broad-leaved angiosperms exhibiting the Péclet effect (*S. tzumu*) (Barbour et al. 2021), the signal fidelity is contingent upon the stability of *L*. The abundant precipitation and highly humid monsoon climate in our study region maintain relatively moderate leaf transpiration rates, thereby strongly restricting the drastic fluctuations in *L* that typically accompany extreme transpiration (Song et al. 2013).

Dual-isotope models typically assume *p*_ex_ to be a constant (0.4) (Guerrieri et al. 2019, Mathias and Thomas 2021); however, recent research indicates that *p*_ex_ decreases as the growing season progresses (Leppä et al. 2026). To quantify the potential confounding effect of this physiological uncertainty on the δ^18^O signal, we performed a sensitivity simulation. By comparing a constant *p*_ex_ scenario (0.4) with a linearly decreasing *p*_ex_ scenario (simulating *p*_ex_ dropping from 0.5 to 0.2 across the growing season), the results showed that Δ^18^O_lw_ and iWUE_mes_ consistently maintained a negative correlation for both *P. massoniana* (*r* = −0.30, *p* < 0.05) and *S. tzumu* (*r* = −0.24, *p* > 0.05) (Figure S4 available as Supplementary Data at *Tree Physiology* Online). While the correlation for *S. tzumu* under the dynamic *p*_ex_ scenario is not statistically significant, the seasonal Δ^18^O_lw_ trajectories from both constant and dynamic *p*_ex_ scenarios remain highly consistent and synchronized (Figure S4c available as Supplementary Data at *Tree Physiology* Online). This demonstrates that the potential dynamic variability of *p*_ex_ does not fundamentally alter the core seasonal covariation trend of the isotopic signals in these tree species.

Additionally, δ^18^O_v_ may introduce additional variability to Δ^18^O_lw_, which confounds the *g*_s_ signal (Gat et al. 2001, Munksgaard et al. 2020, Lin et al. 2022). To evaluate the degree of interference from this environmental noise, we incorporated monthly δ^18^O_v_ and δ^18^O_p_ data simulated by the LMDZ4 model (2007–2010), alongside empirical measurements (2011–2014, including data derived from Li et al. 2020*a*), for auxiliary verification. The model’s capacity to capture the seasonal dynamics of local precipitation isotopes has been validated by comparing the LMDZ4-simulated data with measured δ^18^O_p_ near the sampling site, revealing highly similar seasonal fluctuation characteristics (Figure S5 available as Supplementary Data at *Tree Physiology* Online). The analysis reveals that in the humid East Asian monsoon region, δ^18^O_v_ is highly and positively correlated with precipitation δ^18^O_p_, which serves as the reference source water (*r* = 0.58, *p* < 0.01), indicating that the two maintain an isotopic equilibrium state on a seasonal scale (Figure S5 available as Supplementary Data at *Tree Physiology* Online). Given that Δ^18^O_lw_ in this study is the enrichment value calculated relative to δ^18^O_p_ (Eqs (8) and (9)), this synchronous evolution of water vapor and precipitation isotopes computationally offsets a large portion of the physical interference caused by background δ^18^O_v_ fluctuations. Although the residual effects of δ^18^O_v_ cannot be completely excluded, this relative metric preserves the underlying stomatal physiological signal more effectively than the raw tree-ring δ^18^O.

Finally, it must be acknowledged that there are inherent methodological limitations in attempting to completely decouple the pure *g*_s_ signal from strong monsoon climate noise. In our study, tree-ring δ^18^O was significantly correlated with temperature and RH (Table S5 available as Supplementary Data at *Tree Physiology* Online), indicating unavoidable interference from the background noise of environmental fluctuations (Lin et al. 2022, Carter et al. 2025). Nevertheless, Carter et al. (2025) also noted that despite the methodological uncertainties of solely relying on δ^18^O to infer *g*_s_, the conclusion drawn by Mathias and Thomas (2021) and Guerrieri et al. (2019) using such methods—that ‘*g*_s_ has not significantly changed at most sites’—may still hold true in real-world ecology. Based on plant physiological principles, it is reasonable to deduce that when water is not a limiting factor, trees will avoid reducing *g*_s_ to maintain the intercellular CO_2_ supply, thereby ensuring photosynthetic assimilation. This physiological mechanism aligns well with our empirical inference: in the humid East Asian monsoon region, the seasonal dynamics of iWUE are primarily driven by seasonal variations in *A*, with *g*_s_ playing only a secondary role.

### CO_2_ and temperature jointly determine seasonal iWUE patterns through photosynthetic regulation

On interannual scales, numerous studies have demonstrated that the CO_2_ fertilization effect is the primary driver of long-term iWUE increases (Guerrieri et al. 2019, Mathias and Thomas 2021, Treml et al. 2022, Mathias et al. 2023). At the seasonal scale, this study also found a highly significant positive correlation between atmospheric CO_2_ concentration (*C*_a_) and iWUE (Figure 4; Table S2 available as Supplementary Data at *Tree Physiology* Online). To rule out the ‘mathematical background forcing’ effect caused by *C*_a_ serving as an explicit multiplier in the calculation formula, we used a null-hypothesis model to confirm that this seasonal increase is fundamentally driven by active physiological regulation, rather than being a mere algebraic artifact (Figure S3 available as Supplementary Data at *Tree Physiology* Online).

Multiple linear regression analysis indicated that *C*_a_ was an environmental factor that significantly contributed to the variations in iWUE for both tree species (Figure 5a and b; Tables S3 and S4 available as Supplementary Data at *Tree Physiology* Online). Controlled experimental studies have confirmed that elevated CO_2_, as the primary substrate for photosynthesis, directly enhances *A* while suppressing *g*_s_, collectively increasing iWUE (Bert et al. 1997, Ainsworth and Rogers 2007). In contrast, humidity-related variables (*P*, RH, VPD) had weak effects on iWUE (Figure 4; Table S2 available as Supplementary Data at *Tree Physiology* Online), indicating minimal stomatal limitation under humid conditions and further underscoring the dominant role of *A*. As the main substrate for photosynthesis, CO_2_ directly regulates photosynthetic activity, establishing *C*_a_ as the dominant factor controlling seasonal iWUE through *A*.

This study found that, on a seasonal scale, the iWUE of both tree species was generally significantly negatively correlated with temperature (Figure 4; Table S2 available as Supplementary Data at *Tree Physiology* Online). This is consistent with the observations of Bing et al. (2022) and Grossnickle et al. (2005), but differs from previous studies reporting a positive correlation between high temperature and iWUE (Seibt et al. 2008, Battipaglia et al. 2014). Multiple linear regression analysis further indicated that temperature is one of the key factors driving this seasonal variation (Figure 5a and b; Tables S3 and S4 available as Supplementary Data at *Tree Physiology* Online).

Notably, unlike the monotonic temperature response of *P. massoniana* (Figure 4e), the response of *S. tzumu* iWUE to temperature is not a simple monotonic decrease, but rather exhibits a biphasic characteristic (Figure 4f, as indicated by the red fitted line). From February to April, when temperatures are below 20 °C, the change in iWUE is relatively gradual, and its relationship with temperature is relatively weak (with a slope of only −0.31, Figure 4f). During this period, moisture conditions are relatively abundant, and a moderate temperature increase may promote the *A* while simultaneously driving a corresponding increase in *g*_s_ to support carbon assimilation (Li et al. 2020b). The coordinated changes in *A* and *g*_s_ likely maintain iWUE at a relatively stable level.

According to the temperature response theory of photosynthesis, the leaf photosynthetic rate typically follows a hump-shaped curve, increasing with temperature below the optimum temperature (*T*_opt_) and decreasing once *T*_opt_ is exceeded. For subtropical tree species, *T*_opt_ is generally considered to be within the range of 25–35 °C (Li et al. 2020b). In this study, when the temperature exceeded 20 °C, the *S. tzumu* iWUE showed a distinct downward trend (reaching a slope of −1.51, Figure 4f), suggesting that 20 °C may approach or exceed the optimal temperature threshold for photosynthesis in this species on a seasonal scale. This decline likely reflects the inhibition of photosynthesis by high temperatures and its ‘decoupling’ from stomatal behavior. On one hand, high temperatures may lead to the downregulation of the maximum Rubisco carboxylation rate (*V*_cmax_) and significantly increase the photorespiratory CO_2_ compensation point (Γ*) (Brooks and Farquhar 1985, Bernacchi et al. 2001, Walker et al. 2017, Dusenge et al. 2019), thereby exerting an inhibitory effect on the *A*. On the other hand, under continuous heat stress, trees may tend to maintain relatively high *g*_s_ to sustain transpirational cooling and avoid thermal damage, rather than closing stomata synchronously with the decrease in *A*, thus leading to the decoupling of photosynthesis and stomatal behavior (Wang et al. 2026). Taken together, the observed decline in iWUE during the high-temperature season in this study may imply that photosynthesis is limited under high-temperature conditions while accompanied by high transpirational water loss; this speculation is consistent with the conclusions of Bing et al. (2022).

A key limitation of this study is the fact that intra-annual tree-ring dating remains approximate. Future research should combine continuous micro-coring sampling, high-resolution radial tree growth monitoring networks and simultaneous ecophysiological measurements to accurately determine cambial activity periods. Such approaches would significantly advance our understanding of the coupling between iWUE dynamics and tree carbon–water balance.

## Conclusions

This study reveals that in the humid East Asian monsoon region, the seasonal variation in iWUE is predominantly governed by photosynthetic rate (*A*), establishing a ‘*A*-dominated’ regulatory mechanism distinct from the ‘stomata-dominated’ pattern observed in arid regions. Although *P. massoniana* and *S. tzumu* differ in their specific response strategies, the consistent seasonal iWUE pattern and its relationship with environmental factors jointly indicate that atmospheric CO_2_ concentration and temperature are the key climatic drivers of iWUE variation. Humidity-related variables exhibit weak influences, confirming minimal stomatal limitation under humid conditions. The summer decline in iWUE is primarily attributable to the combined constraints of supra-optimal high temperatures (leading to photosynthetic limitation and stomatal decoupling) and limited CO_2_ availability.

In humid forest ecosystems, carbon acquisition rather than water conservation is likely the key limiting factor for tree growth. Under future climate warming, even in moisture-sufficient regions, rising temperatures may directly suppress photosynthesis, thereby restricting tree growth and carbon accumulation and thus weakening forest carbon sink capacity. However, we acknowledge that current isotope-based physiological modeling entails inherent uncertainties, particularly regarding the background noise generated by environmental fluctuations. Therefore, it is essential for future forest carbon sink models to fully incorporate the seasonal dynamics of CO_2_ supply and its interaction with temperature, and to couple high-resolution isotopic analysis with *in situ* continuous monitoring to constrain these model uncertainties, so as to avoid overestimating the carbon sequestration potential of forests in climate change projections.

## Supplementary Material

He_etal_SI_2026_3_18_tpag061

## Data Availability

The dataset is available from the World Data Center for Geophysics, Beijing (https://doi.org/10.12197/2025GA041).

## References

[ref1] Adams MA, Buckley TN, Turnbull TL (2020) Diminishing CO_2_-driven gains in water-use efficiency of global forests. Nat Clim Chang 10:466–471. 10.1038/s41558-020-0747-7.

[ref2] Ainsworth EA, Rogers A (2007) The response of photosynthesis and stomatal conductance to rising [CO_2_]: mechanisms and environmental interactions. Plant Cell Environ 30:258–270. 10.1111/j.1365-3040.2007.01641.x. 17263773

[ref3] An W, Xu C, Liu X, Tan N, Sano M, Li M, Shao X, Nakatsuka T, Guo Z (2019) Specific response of earlywood and latewood δ^18^O from the east and west of Mt. Qomolangma to the Indian summer monsoon. Sci Total Environ 689:99–108. 10.1016/j.scitotenv.2019.06.268. 31271994

[ref4] Andreu-Hayles L, Lévesque M, Guerrieri R, Siegwolf RT, Körner C (2022) Limits and strengths of tree-ring stable isotopes. In: Siegwolf RTW, Brooks JR, Roden J, Saurer M (eds) Stable isotopes in tree rings: inferring physiological, climatic and environmental responses. Springer International Publishing, Cham (Switzerland), pp 399–428.

[ref7] Barbour MM, Walcroft AS, Farquhar GD (2002) Seasonal variation in δ^13^C and δ^18^O of cellulose from growth rings of *Pinus radiata*. Plant Cell Environ 25:1483–1499. 10.1046/j.0016-8025.2002.00931.x.

[ref6] Barbour MM, Roden JS, Farquhar GD, Ehleringer JR (2004) Expressing leaf water and cellulose oxygen isotope ratios as enrichment above source water reveals evidence of a Péclet effect. Oecologia 138:426–435. 10.1007/s00442-003-1449-3. 14666420

[ref5] Barbour MM, Loucos KE, Lockhart EL, Shrestha A, McCallum D, Simonin KA, Song X, Griffani DS, Farquhar GD (2021) Can hydraulic design explain patterns of leaf water isotopic enrichment in C_3_ plants? Plant Cell Environ 44:432–444. 10.1111/pce.13943. 33175397

[ref8] Battipaglia G, De Micco V, Brand WA, Saurer M, Aronne G, Linke P, Cherubini P (2014) Drought impact on water use efficiency and intra-annual density fluctuations in *Erica arborea* on Elba (Italy). Plant Cell Environ 37:382–391. 10.1111/pce.12160. 23848555

[ref9] Berkelhammer M, Stott LD (2009) Modeled and observed intra-ring δ^18^O cycles within late Holocene bristlecone pine tree samples. Chem Geol 264:13–23. 10.1016/j.chemgeo.2009.02.010.

[ref10] Bernacchi CJ, Singsaas EL, Pimentel C, Portis AR, Long SP (2001) Improved temperature response functions for models of Rubisco-limited photosynthesis. Plant Cell Environ 24:253–259. 10.1111/j.1365-3040.2001.00668.x.

[ref11] Bert D, Leavitt SW, Dupouey JL (1997) Variations of wood δ^13^C and water-use efficiency of *Abies alba* during the last century. Ecology 78:1588–1596. 10.1890/0012-9658(1997)078[1588:VOWCAW]2.0.CO;2.

[ref12] Bing X, Fang K, Gong X et al. (2022) The intra-annual intrinsic water use efficiency dynamics based on an improved model. Clim Change 172:16. 10.1007/s10584-022-03368-1.

[ref13] Bögelein R, Hassdenteufel M, Thomas FM, Werner W (2012) Comparison of leaf gas exchange and stable isotope signature of water-soluble compounds along canopy gradients of co-occurring Douglas-fir and European beech. Plant Cell Environ 35:1245–1257. 10.1111/j.1365-3040.2012.02486.x. 22292498

[ref14] Brooks A, Farquhar GD (1985) Effect of temperature on the CO_2_/O_2_ specificity of ribulose-1,5-bisphosphate carboxylase/oxygenase and the rate of respiration in the light-estimates from gas-exchange measurements on spinach. Planta 165:397–406. 10.1007/BF00392238. 24241146

[ref15] Carnicer J, Brbeta A, Sperlich D, Coll M, Penuelas J (2013) Contrasting trait syndromes in angiosperms and conifers are associated with different responses of tree growth to temperature on a large scale. Front Plant Sci 4:409. 10.3389/fpls.2013.00409. 24146668 PMC3797994

[ref16] Carter I, Brienen R, Gloor M (2025) Can oxygen isotopes in tree rings be used to detect stomatal responses to global change? Glob Chang Biol 31:e70604. 10.1111/gcb.70604. 41239855 PMC12619108

[ref17] Cheesman AW, Cernusak LA (2017) Infidelity in the outback: climate signal recorded in Δ^18^O of leaf but not branch cellulose of eucalypts across an Australian aridity gradient. Tree Physiol 37:554–564. 10.1093/treephys/tpw121. 28008083

[ref18] Chen H, Liu J, Jiang J, Sun Y, Dong X, Fu A (2020) A study on flowering and fruit development of *Sassafras tzumu*. For Res 33:148–154 (in Chinese with English abstract). 10.13275/j.cnki.lykxyj.2020.06.018.

[ref19] Choat B, Jansen S, Brodribb TJ et al. (2012) Global convergence in the vulnerability of forests to drought. Nature 491:752–755. 10.1038/nature11688. 23172141

[ref20] Duan W, Ruan J, Luo W et al. (2016) The transfer of seasonal isotopic variability between precipitation and drip water at eight caves in the monsoon regions of China. Geochim Cosmochim Acta 183:250–266. 10.1016/j.gca.2016.03.037.

[ref21] Dusenge ME, Duarte AG, Way DA (2019) Plant carbon metabolism and climate change: elevated CO_2_ and temperature impacts on photosynthesis, photorespiration and respiration. New Phytol 221:32–49. 10.1111/nph.15283. 29983005

[ref23] Farquhar GD, Lloyd J (1993) Carbon and oxygen isotope effects in the exchange of carbon dioxide between terrestrial plants and the atmosphere. In: Ehleringer JR, Hall AE, Farquhar GD (eds) Stable isotopes and plant carbon-water relations. Academic Press, San Diego, CA, pp 47–70.

[ref24] Farquhar GD, O’Leary MH, Berry JA (1982) On the relationship between carbon isotope discrimination and the inter-cellular carbon-dioxide concentration in leaves. Aust J Plant Physiol 9:121–137. 10.1071/PP9820121.

[ref22] Farquhar GD, Cernusak LA, Barnes B (2007) Heavy water fractionation during transpiration. Plant Physiol 143:11–18. 10.1104/pp.106.093278. 17210909 PMC1761995

[ref25] Fichtler E, Helle G, Worbes M (2010) Stable-carbon isotope time series from tropical tree rings indicate a precipitation signal. Tree Ring Res 66:35–49. 10.3959/2008-20.1.

[ref26] Gat J, Mook W, Meijer H (2001) Environmental isotopes in the hydrological cycle: principles and applications, Vol. 2, Atmospheric water. UNESCO-IAEA, Vienna, Austria.

[ref27] Grossiord C, Buckley TN, Cernusak LA, Novick KA, Poulter B, Siegwolf RTW, Sperry JS, McDowell NG (2020) Plant responses to rising vapor pressure deficit. New Phytol 226:1550–1566. 10.1111/nph.16485. 32064613

[ref28] Grossnickle SC, Fan S, Russell JH (2005) Variation in gas exchange and water use efficiency patterns among populations of western redcedar. Trees 19:32–42. 10.1007/s00468-004-0360-9.

[ref29] Guerrieri R, Belmecheri S, Ollinger SV et al. (2019) Disentangling the role of photosynthesis and stomatal conductance on rising forest water-use efficiency. Proc Natl Acad Sci U S A 116:16909–16914. 10.1073/pnas.1905912116. 31383758 PMC6708355

[ref30] He Y, Fan G, Zhang X, Gao D, Hu B (2012) Vegetation phenology monitoring and spatio-temporal dynamics in Zhejiang Province in past 10 years. Chin Agric Sci Bull 28:117–124 (in Chinese with English abstract). 10.11924/j.issn.1000-6850.2012-0078.

[ref31] Helle G, Schleser GH (2004) Beyond CO_2_-fixation by Rubisco: an interpretation of ^13^C/^12^C variations in tree rings from novel intra-seasonal studies on broad-leaf trees. Plant Cell Environ 27:367–380. 10.1111/j.0016-8025.2003.01159.x.

[ref32] Hourdin F, Musat I, Bony S et al. (2006) The LMDZ4 general circulation model: climate performance and sensitivity to parametrized physics with emphasis on tropical convection. Clim Dyn 27:787–813. 10.1007/s00382-006-0158-0.

[ref34] Huang R, Zhu H, Liu X, Liang E, Grießinger J, Wu G, Li X, Bräuning A (2017) Does increasing intrinsic water use efficiency (iWUE) stimulate tree growth at natural alpine timberline on the southeastern Tibetan plateau? Global Planet Change 148:217–226. 10.1016/j.gloplacha.2016.11.017.

[ref33] Huang R, Zhu H, Liang E et al. (2019) Temperature signals in tree-ring oxygen isotope series from the northern slope of the Himalaya. Earth Planet Sci Lett 506:455–465. 10.1016/j.epsl.2018.11.002.

[ref35] Jiang A, Jiang J, Liu J (2016) Responses of leaf traits of *Sassafras tzumu* (Hemsl.) Hemsl. along an altitudinal gradient. Chin J Ecol 35:1467–1474 (in Chinese with English abstract). 10.13292/j.1000-4890.201606.017.

[ref36] Kagawa A, Battipaglia G (2022) Post-photosynthetic carbon, oxygen and hydrogen isotope signal transfer to tree rings—how timing of cell formations and turnover of stored carbohydrates affect intra-annual isotope variations. In: Siegwolf RTW, Brooks JR, Roden J, Saurer M (eds) Stable isotopes in tree rings: inferring physiological, climatic and environmental responses. Springer International Publishing, Cham, Switzerland, pp 429–462.

[ref37] Kagawa A, Sugimoto A, Maximov TC (2006) Seasonal course of translocation, storage and remobilization of δ^13^C pulse-labeled photoassimilate in naturally growing *Larix gmelinii* saplings. New Phytol 171:793–804. 10.1111/j.1469-8137.2006.01780.x. 16918550

[ref38] Klein T, Shpringer I, Fikler B, Elbaz G, Cohen S, Yakir D (2013) Relationships between stomatal regulation, water-use, and water-use efficiency of two coexisting key Mediterranean tree species. For Ecol Manage 302:34–42. 10.1016/j.foreco.2013.03.044.

[ref39] Leppä K, Szejner P, Angove C et al. (2026) Interpreting inter- and intra-annual environmental signals in tree-ring δ^18^O using isotope-enabled modeling. Tree Physiol 46:tpag026. 10.1093/treephys/tpag026. 41714183 PMC13064660

[ref40] Li F, Xiao J, Chen J, Ballantyne A, Jin K, Li B, Abraha M, John R (2023) Global water use efficiency saturation due to increased vapor pressure deficit. Science 381:672–677. 10.1126/science.adf5041. 37561856

[ref41] Li Y, An W, Pang H, Wu S-Y, Tang Y, Zhang W, Hou S (2020 *a*) Variations of stable isotopic composition in atmospheric water vapor and their controlling factors—a 6-year continuous sampling study in Nanjing, Eastern China. J Geophys Res Atmos 125:e2019JD031697. 10.1029/2019JD031697.

[ref42] Li Y, Xu Y, Li Y et al. (2020 *b*) Warming effects on morphological and physiological performances of four subtropical montane tree species. Ann For Sci 77:2. 10.1007/s13595-019-0910-3.

[ref43] Lin W, Barbour MM, Song X (2022) Do changes in tree-ring δ^18^O indicate changes in stomatal conductance? New Phytol 236:803–808. 10.1111/nph.18431. 36200332

[ref44] Ma WT, Tcherkez G, Wang XM, Schäufele R, Schnyder H, Yang Y, Gong XY (2021) Accounting for mesophyll conductance substantially improves δ^13^C-based estimates of intrinsic water-use efficiency. New Phytol 229:1326–1338. 10.1111/nph.16958. 32984961

[ref45] Martínez-Sancho E, Treydte K, Lehmann MM, Rigling A, Fonti P (2022) Drought impacts on tree carbon sequestration and water use-evidence from intra-annual tree-ring characteristics. New Phytol 236:58–70. 10.1111/nph.18224. 35576102 PMC9542003

[ref47] Mathias JM, Thomas RB (2021) Global tree intrinsic water use efficiency is enhanced by increased atmospheric CO_2_ and modulated by climate and plant functional types. Proc Natl Acad Sci U S A 118:e2014286118. 10.1073/pnas.2014286118. 33558233 PMC7896309

[ref46] Mathias JM, Smith KR, Lantz KE, Allen KT, Wright MJ, Sabet A, Anderson-Teixeira KJ, Thomas RB (2023) Differences in leaf gas exchange strategies explain *Quercus rubra* and *Liriodendron tulipifera* intrinsic water use efficiency responses to air pollution and climate change. Glob Chang Biol 29:3449–3462. 10.1111/gcb.16673. 36897273

[ref48] McAdam SA, Brodribb TJ (2015) The evolution of mechanisms driving the stomatal response to vapor pressure deficit. Plant Physiol 167:833–843. 10.1104/pp.114.252940. 25637454 PMC4348763

[ref49] McCarroll D, Whitney M, Young GHF, Loader NJ, Gagen MH (2017) A simple stable carbon isotope method for investigating changes in the use of recent versus old carbon in oak. Tree Physiol 37:1021–1027. 10.1093/treephys/tpx030. 28338989

[ref50] Michelot A, Simard S, Rathgeber C, Dufrene E, Damesin C (2012) Comparing the intra-annual wood formation of three European species (*Fagus sylvatica*, *Quercus petraea* and *Pinus sylvestris*) as related to leaf phenology and non-structural carbohydrate dynamics. Tree Physiol 32:1033–1045. 10.1093/treephys/tps052. 22718524

[ref51] Miyahara AAL, Locosselli GM (2024) Challenges and advances in intra-annual tree-ring stable isotope research, a systematic review. Dendrochronologia 85:126218. 10.1016/j.dendro.2024.126218.

[ref52] Mo L, Zohner CM, Reich PB et al. (2023) Integrated global assessment of the natural forest carbon potential. Nature 624:92–101. 10.1038/s41586-023-06723-z. 37957399 PMC10700142

[ref53] Morais MC, Cabral JA, Gonçalves B (2021) Seasonal variation in the leaf physiology of co-occurring invasive (*Hakea sericea*) and native (*Pinus pinaster*) woody species in a Mediterranean-type ecosystem. For Ecol Manage 480:118662. 10.1016/j.foreco.2020.118662.

[ref54] Munksgaard NC, Zwart C, Haig J, Cernusak LA, Bird MI (2020) Coupled rainfall and water vapour stable isotope time series reveal tropical atmospheric processes on multiple timescales. Hydrol Process 34:111–124. 10.1002/hyp.13576.

[ref55] Murray FW (1967) On the computation of saturation vapor pressure. J Appl Meteorol Climatol 6:203–204.

[ref56] O’Brien RM (2007) A caution regarding rules of thumb for variance inflation factors. Qual Quant 41:673–690. 10.1007/s11135-006-9018-6.

[ref57] Ogée J, Barbour MM, Wingate L et al. (2009) A single-substrate model to interpret intra-annual stable isotope signals in tree-ring cellulose. Plant Cell Environ 32:1071–1090. 10.1111/j.1365-3040.2009.01989.x. 19422614

[ref58] Ogle N, McCormac FG (1994) High-resolution δ^13^C measurements of oak show a previously unobserved spring depletion. Geophys Res Lett 21:2373–2375. 10.1029/94GL02484.

[ref59] Portarena S, Farinelli D, Famiani F, Cinosi N, Traini C, Rezaei N, Brugnoli E (2024) Differential tolerance to summer stress conditions in two olive cultivars using the dendro-isotopic approach. Dendrochronologia 84:126182. 10.1016/j.dendro.2024.126182.

[ref60] Reed CC, Ballantyne AP, Cooper LA, Sala A (2018) Limited evidence for CO_2_-related growth enhancement in northern Rocky Mountain lodgepole pine populations across climate gradients. Glob Chang Biol 24:3922–3937. 10.1111/gcb.14165. 29658158

[ref61] Ren W, Tian L, Querejeta JI (2024) Tight coupling between leaf δ^13^C and N content along leaf ageing in the N_2_-fixing legume tree black locust (*Robinia pseudoacacia* L.). Physiol Plant 176:e14235. 10.1111/ppl.14235. 38472162

[ref62] Roden JS, Lin GG, Ehleringer JR (2000) A mechanistic model for interpretation of hydrogen and oxygen isotope ratios in tree-ring cellulose. Geochim Cosmochim Acta 64:21–35. 10.1016/S0016-7037(99)00195-7.

[ref63] Rubio-Cuadrado Á, Montes F, Pardos M, Camarero JJ (2024) Differences in hydrological niche and tree size explain growth resilience to drought in three Mediterranean oaks. Agric For Meteorol 359:110291. 10.1016/j.agrformet.2024.110291.

[ref64] Scheidegger Y, Saurer M, Bahn M, Siegwolf R (2000) Linking stable oxygen and carbon isotopes with stomatal conductance and photosynthetic capacity: a conceptual model. Oecologia 125:350–357. 10.1007/s004420000466. 28547329

[ref65] Schubert BA, Timmermann A (2015) Reconstruction of seasonal precipitation in Hawai’i using high-resolution carbon isotope measurements across tree rings. Chem Geol 417:273–278. 10.1016/j.chemgeo.2015.10.013.

[ref66] Seibt U, Rajabi A, Griffiths H, Berry JA (2008) Carbon isotopes and water use efficiency: sense and sensitivity. Oecologia 155:441–454. 10.1007/s00442-007-0932-7. 18224341

[ref67] Shi C, Masson-Delmotte V, Risi C et al. (2011) Sampling strategy and climatic implications of tree-ring stable isotopes on the southeast Tibetan Plateau. Earth Planet Sci Lett 301:307–316. 10.1016/j.epsl.2010.11.014.

[ref68] Simard S, Giovannelli A, Treydte K, Traversi ML, King GM, Frank D, Fonti P (2013) Intra-annual dynamics of non-structural carbohydrates in the cambium of mature conifer trees reflects radial growth demands. Tree Physiol 33:913–923. 10.1093/treephys/tpt075. 24128848

[ref69] Song X, Barbour MM, Farquhar GD, Vann DR, Helliker BR (2013) Transpiration rate relates to within- and across-species variations in effective path length in a leaf water model of oxygen isotope enrichment. Plant Cell Environ 36:1338–1351. 10.1111/pce.12063. 23305086

[ref70] Song X, Clark KS, Helliker BR (2014) Interpreting species-specific variation in tree-ring oxygen isotope ratios among three temperate forest trees. Plant Cell Environ 37:2169–2182. 10.1111/pce.12317. 24588709

[ref71] Sternberg L, Ellsworth PFV (2011) Divergent biochemical fractionation, not convergent temperature, explains cellulose oxygen isotope enrichment across latitudes. PLoS One 6:e28040. 10.1371/journal.pone.0028040. 22132203 PMC3221677

[ref72] Tang Y, Sahlstedt E, Young G, Schiestl-Aalto P, Saurer M, Kolari P, Jyske T, Bäck J, Rinne-Garmston KT (2023) Estimating intraseasonal intrinsic water-use efficiency from high-resolution tree-ring δ^13^C data in boreal Scots pine forests. New Phytol 237:1606–1619. 10.1111/nph.18649. 36451527 PMC10108005

[ref73] Treml V, Tumajer J, Jandová K et al. (2022) Increasing water-use efficiency mediates effects of atmospheric carbon, sulfur, and nitrogen on growth variability of central European conifers. Sci Total Environ 838:156483. 10.1016/j.scitotenv.2022.156483. 35675888

[ref74] Vaganov EA, Schulze ED, Skomarkova MV, Knohl A, Brand WA, Roscher C (2009) Intra-annual variability of anatomical structure and δ^13^C values within tree rings of spruce and pine in alpine, temperate and boreal Europe. Oecologia 161:729–745. 10.1007/s00442-009-1421-y. 19653008 PMC2744769

[ref75] Walker AP, De Kauwe MG, Bastos A et al. (2021) Integrating the evidence for a terrestrial carbon sink caused by increasing atmospheric CO_2_. New Phytol 229:2413–2445. 10.1111/nph.16866. 32789857

[ref76] Walker BJ, Orr DJ, Carmo-Silva E, Parry MAJ, Bernacchi CJ, Ort DR (2017) Uncertainty in measurements of the photorespiratory CO_2_ compensation point and its impact on models of leaf photosynthesis. Photosynth Res 132:245–255. 10.1007/s11120-017-0369-8. 28382593 PMC5443873

[ref77] Wang L, Liu H, Shi L et al. (2024) Water use strategies determine divergent growth trends of spruce and juniper on the southeastern Tibetan plateau. For Ecosyst 11:100248. 10.1016/j.fecs.2024.100248.

[ref78] Wang Z, Slot M, Wang C (2026) Decoupling of stomatal conductance, transpiration and photosynthesis in terrestrial plants under elevated temperature: a meta-analysis. Nat Commun 17:1528. 10.1038/s41467-025-68250-x. 41507225 PMC12891522

[ref79] Wilson AT, Grinsted MJ (1975) Palaeotemperatures from tree rings and the D/H ratio of cellulose as a biochemical thermo-meter. Nature 257:387–388. 10.1038/257387a0.

[ref80] Xia L (2016) Stable isotope tracing of atmospheric CO_2_ background concentration and source-sink characteristics at Shangdianzi Station, Beijing and Lin’an Station. Zhejiang [dissertation], Chinese Academy of Meteorological Sciences, Beijing, China.

[ref81] Xia L, Zhou L, Tans PP, Liu L, Zhang G, Wang H, Luan T (2015) Atmospheric CO_2_ and its δ^13^C measurements from flask sampling at Lin’an regional background station in China. Atmos Environ 117:220–226. 10.1016/j.atmosenv.2015.07.008.

[ref83] Xu C, Sano M, Nakatsuka T (2011) Tree-ring cellulose δ^18^O of *Fokienia hodginsii* in Northern Laos: a promising proxy to reconstruct ENSO. J Geophys Res 116:D24109. 10.1029/2011JD016694.

[ref82] Xu C, Huang R, An W, Zhao Q, Zhao Y, Ren J, Liu Y, Guo Z (2024) Tree ring oxygen isotope in Asia. Global Planet Change 232:104348. 10.1016/j.gloplacha.2023.104348.

[ref84] Xu G, Liu X, Belmecheri S, Chen T, Wu G, Wang B, Zeng X, Wang W (2018) Disentangling contributions of CO_2_ concentration and climate to changes in intrinsic water-use efficiency in the arid boreal forest in China’s Altay Mountains. Forests 9:642. 10.3390/f9100642.

[ref85] Xu G, Liu X, Hu J, Dorado-Linan I, Gagen M, Szejner P, Chen T, Trouet V (2022) Intra-annual tree-ring δ^18^O and δ^13^C reveal a trade-off between isotopic source and humidity in moist environments. Tree Physiol 42:2203–2223. 10.1093/treephys/tpac076. 35796563

