# Supplementary material for: Photosynthetic rate dominates the seasonal variation of tree intrinsic water-use efficiency in the humid East Asian Monsoon region
Source: Tree Physiol. 2026 Apr 30;46(6):tpag061. doi: 10.1093/treephys/tpag061 (PMC13252599; doi:10.1093/treephys/tpag061)
Supplement: He_etal_SI_2026_3_18_tpag061 [file he_etal_si_2026_3_18_tpag061.docx]

**Supplementary Information**

Xinyu He^1,2^, Yao Li^1,2^, Ru Huang^3^, Wenling An^1,2^, Kerstin Treydte^4,5^, Qingyu Zhao^1,2^, Junbo Ren^1,2^, Sainan Liu^1,2^, Chenxi Xu^1,2,*^

^1^ State Key Laboratory of Lithospheric and Environmental Coevolution, Institute of Geology and Geophysics, Chinese Academy of Sciences, Beijing 100029, China;

^2^ College of Earth and Planetary Sciences, University of Chinese Academy of Sciences, Beijing 101408, China;

^3^ Department of Environment and Biodiversity, Paris-Lodron-University of Salzburg, Salzburg, 5020, Austria;

^4^ Research Unit Forest and Soil Ecology, Swiss Federal Institute for Forest, Snow and Landscape Research WSL, 8903 Birmensdorf, Switzerland.

^5^ Oeschger Oeschger Centre for Climate Change Research, University of Bern,
3012 Bern, Switzerland

*Corresponding author: Chenxi Xu, [cxxu@mail.iggcas.ac.cn](mailto:cxxu@mail.iggcas.ac.cn)

State Key Laboratory of Lithospheric and Environmental Coevolution, Institute of Geology and Geophysics, Chinese Academy of Sciences, Beijing 100029, China


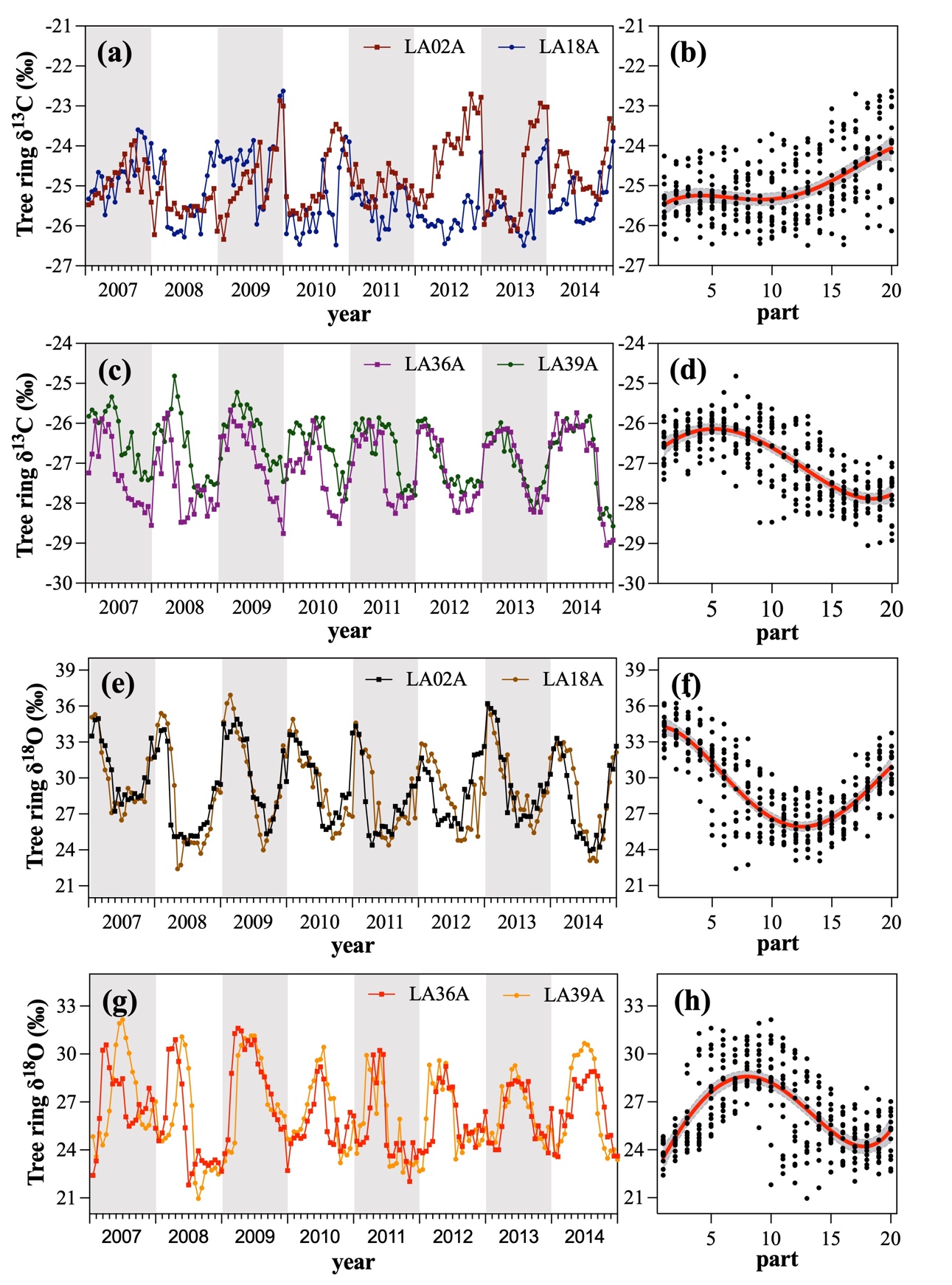


Fig. S1. Seasonal variations in unsynchronized (raw) tree-ring δ^13^C (a, c) and their mean seasonal patterns (b, d) for *P. massoniana* (a, b) and *S. tzumu* (c, d) during the period 2007–2014; Seasonal variations unsynchronized (raw) in tree-ring δ^18^O (e, g) and their mean seasonal patterns (f, h) for *P. massoniana* (e, f) and *S. tzumu* (g, h) during the period 2007–2014.


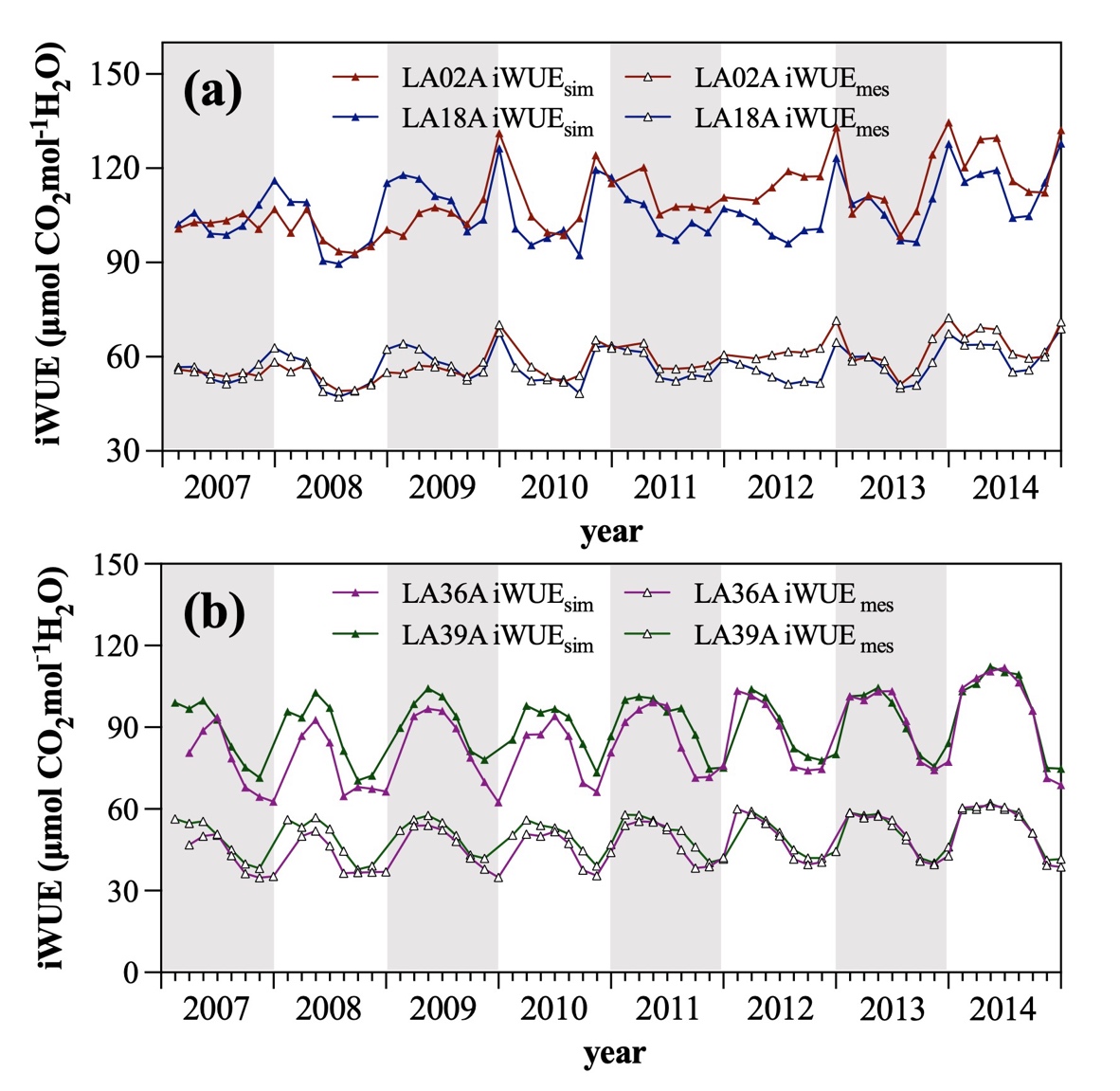


Fig. S2. iWUE estimations in (a) *P. massoniana* and (b) *S. tzumu*. The simplified iWUE_sim_ model neglects g_m_ constraints, whereas the iWUE_mes_ model explicitly incorporates g_m_ limitations.


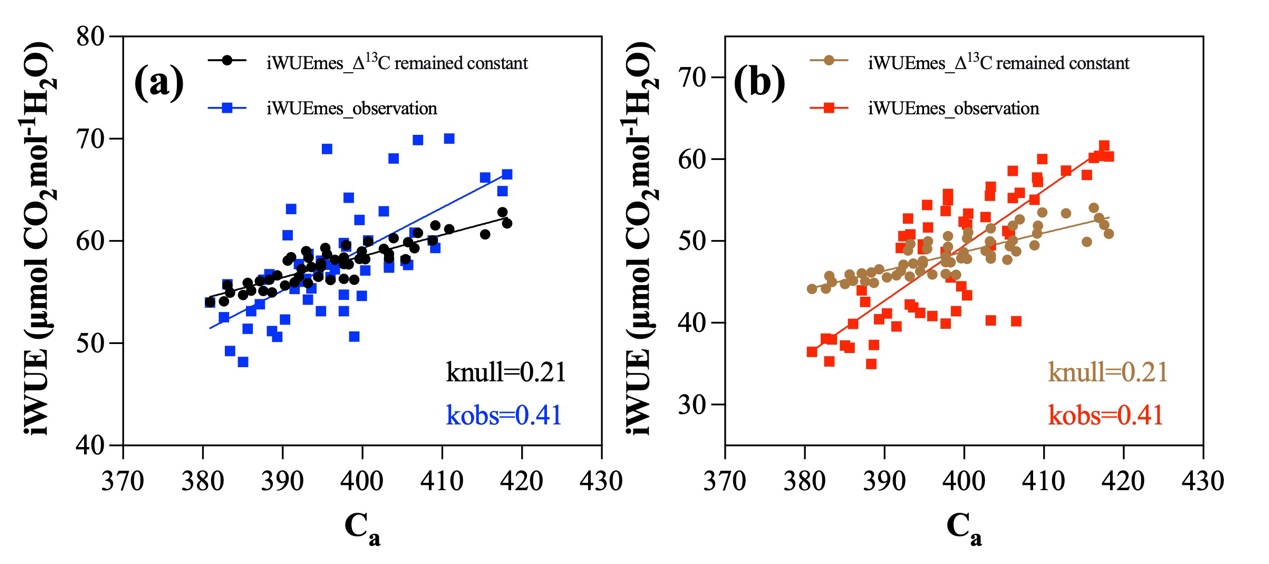


Fig. S3 Comparison between observed slopes (k_obs_) and null-hypothesis model slopes (k_null_) of iWUE in response to atmospheric CO_2_ concentration (C_a_) for *P. massoniana* (a) and *S. tzumu* (b).


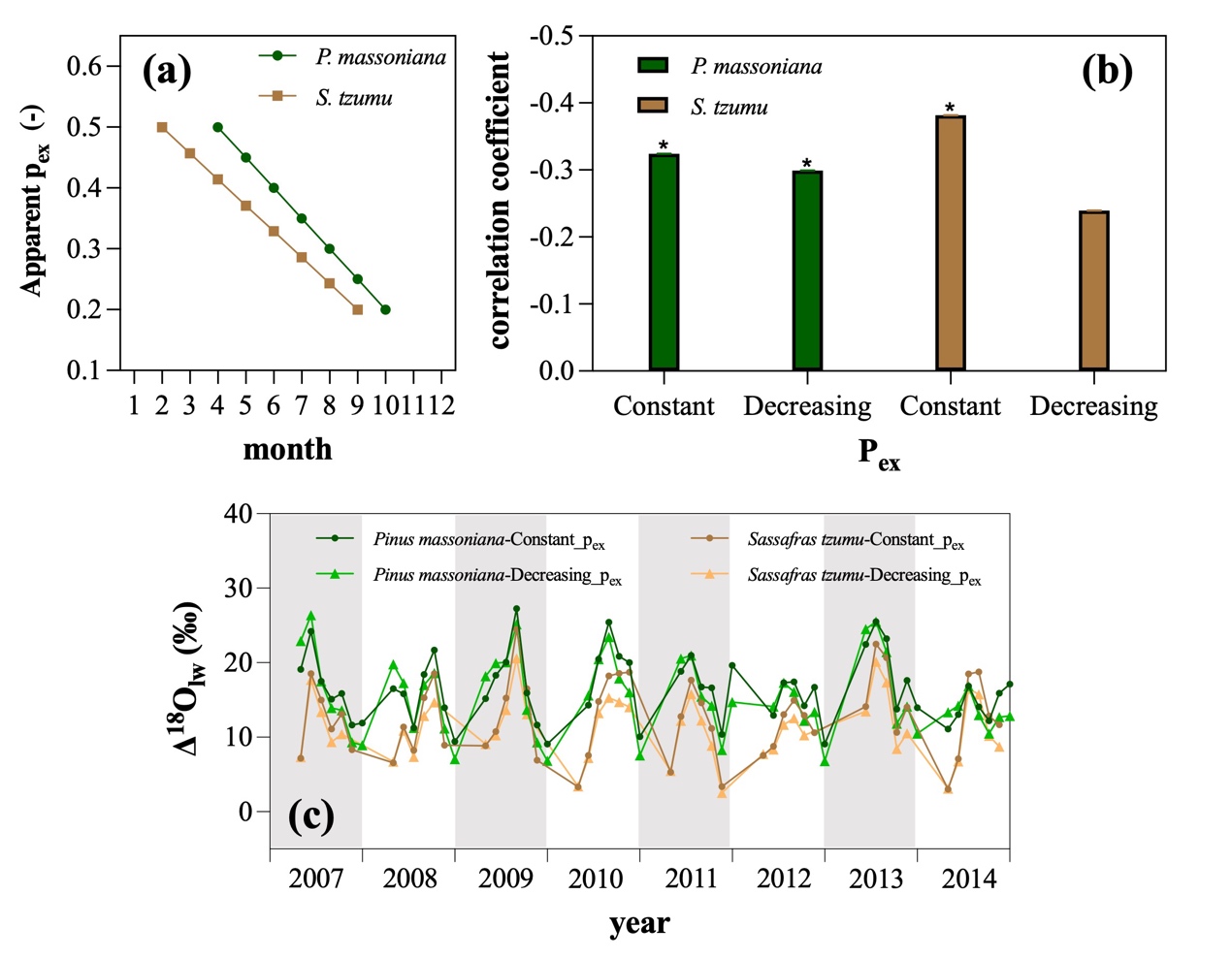
 Fig. S4. Sensitivity analysis evaluating the seasonal variation in the apparent oxygen exchange proportion (p_ex_). (a) Seasonal parameter settings for p_ex_ applied in the simulations, following the observational patterns in Leppä et al. (2026) where p_ex_ decreases linearly from 0.5 to 0.2 over the growing season. (b) Comparison of correlation coefficients between Δ^18^O_lw_ and iWUE_mes_ for *P. massoniana* and *S. tzumu* under constant p_ex_ (0.4) and linearly decreasing p_ex_ scenarios. Asterisks (*) indicate significant correlations (*P* < 0.05). (c) Variations of Δ^18^Olw in *P. massoniana* and *S. tzumu* under constant and linearly decreasing p_ex_ scenarios.


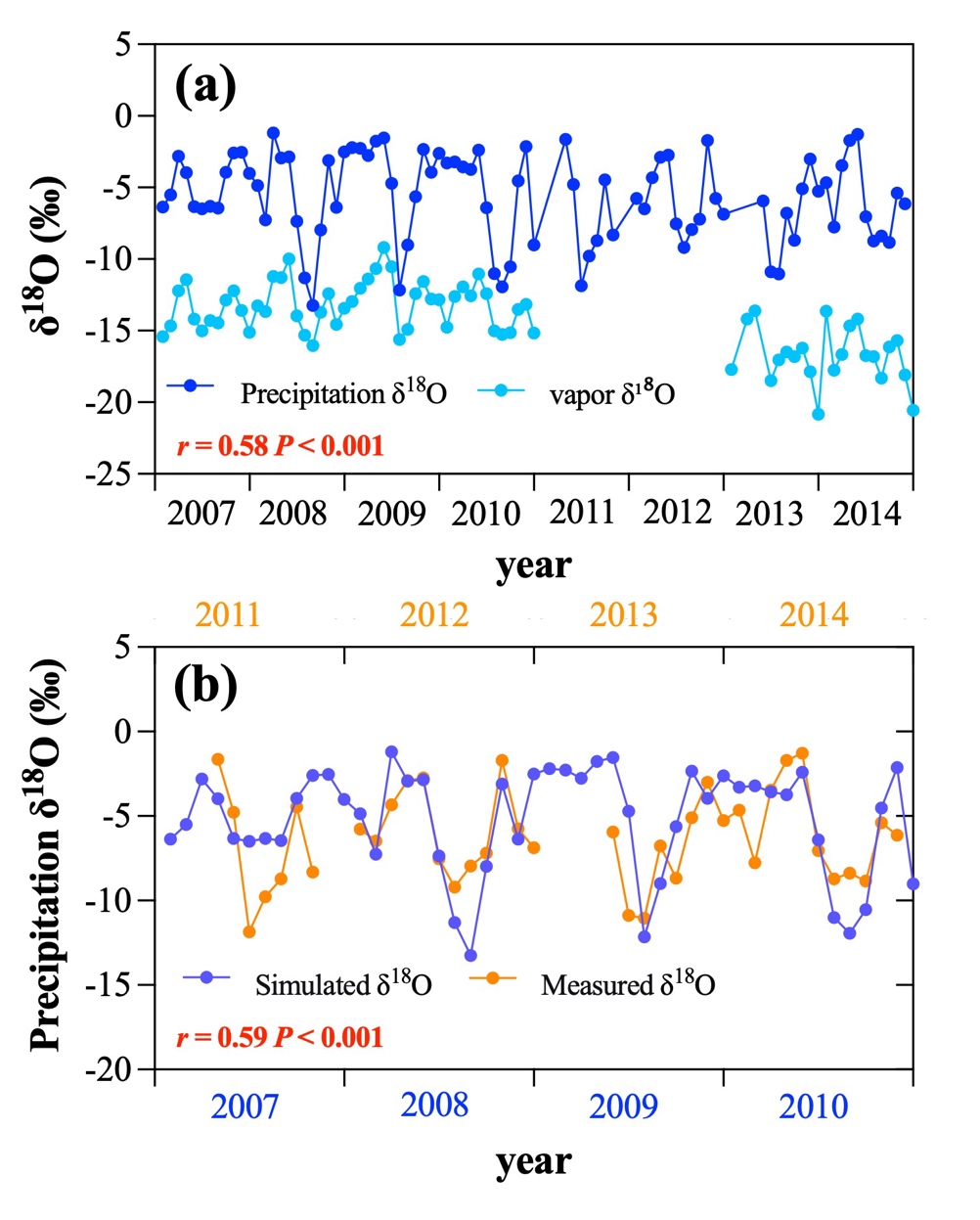


Fig. S5. (a) Seasonal variation and correlation of atmospheric water vapor oxygen isotopes (δ^18^O_v_) and precipitation oxygen isotopes (δ^18^O_p_) from 2007 to 2014. Data for 2007–2010 are simulated by the LMDZ4 model, while data for 2011–2014 are empirical measurements (the measured data for 2013–2014 are derived from Li et al., 2020). (b) Comparison of the seasonal patterns between measured δ^18^O_p_ (2011–2014) near the sampling site and the LMDZ4 simulated data (2007–2010), demonstrating similar seasonal fluctuation characteristics.

Table S1. Dates of the 20 sub-samples of each ring

| Tree species | Part | Date | Tree species | Part | Date |
| --- | --- | --- | --- | --- | --- |
| *P. massoniana* | 1 | Apr 1 - Apr 11 | *S. tzumu* | 1 | Feb 15 - Feb 25 |
|  | 2 | Apr 12 - Apr 22 |  | 2 | Feb 26 - Mar 7 |
|  | 3 | Apr 23 - May 3 |  | 3 | Mar 8 - Mar 18 |
|  | 4 | May 4 - May 13 |  | 4 | Mar 19 - Mar 29 |
|  | 5 | May 14 - May 24 |  | 5 | Mar 30 - Apr 9 |
|  | 6 | May 25-Jun 4 |  | 6 | Apr 10 - Apr 20 |
|  | 7 | Jun 5 - Jun 14 |  | 7 | Apr 21 - May 1 |
|  | 8 | Jun 15 - Jun 25 |  | 8 | May 2 - May 12 |
|  | 9 | Jun 26 - Jul 6 |  | 9 | May 13 - May 23 |
|  | 10 | Jul 7 - Jul 17 |  | 10 | May 24 - Jun 3 |
|  | 11 | Jul 18 - Jul 27 |  | 11 | Jun 4 - Jun 13 |
|  | 12 | Jul 28 - Aug 7 |  | 12 | Jun 14 - Jun 24 |
|  | 13 | Aug 8 - Aug 18 |  | 13 | Jun 25 - Jul 5 |
|  | 14 | Aug 19 - Aug 28 |  | 14 | Jul 6 - Jul 16 |
|  | 15 | Aug 29 - Sep 8 |  | 15 | Jul 17 - Jul 27 |
|  | 16 | Sep 9 - Sep 19 |  | 16 | Jul 28 - Aug 7 |
|  | 17 | Sep 20 - Sep 29 |  | 17 | Aug 8 - Aug 18 |
|  | 18 | Sep 30 - Oct 10 |  | 18 | Aug 19 - Aug 29 |
|  | 19 | Oct 11 - Oct 21 |  | 19 | Aug 30 - Sep 9 |
|  | 20 | Oct 22 - Nov 1 |  | 20 | Sep 10 - Sep 20 |

Table S2. Pearson correlation coefficients iWUE (calculated from tree-ring cellulose δ^13^C) and climatic factors during 2007–2014 for *P. massoniana* (April–October climatic data, *n* = 56) and *S. tzumu* (February–September climatic data, *n* = 64).

|  | *P. massoniana* | *S. tzumu* |
| --- | --- | --- |
| T | -0.57*** | -0.76*** |
| P | -0.33* | -0.22 |
| RH | -0.03 | -0.25 |
| VPD | -0.41** | -0.51*** |
| C_a_ | 0.68*** | 0.79*** |

Significance levels: ****P* < 0.001, ***P* < 0.01, *P < 0.05.

| Table S3. Results of multiple linear regression analysis for *P. massoniana* (April–October climatic data, *n*=56) | | | | | | | |
| --- | --- | --- | --- | --- | --- | --- | --- |
|  | Unstandardized Coefficients | | Standardized Coefficients | *t* | *P* | Collinearity Diagnostics | |
|  | *B* | Std. Error | *Beta* |  |  | VIF | Tolerance |
| Constant | 0.000 | 0.091 | - | 0.000 | 1.000 | - | - |
| C_a_ | 0.537 | 0.101 | 0.537 | 5.312 | 0.000** | 1.221 | 0.819 |
| VPD | -0.115 | 0.117 | -0.115 | -0.982 | 0.331 | 1.625 | 0.615 |
| T | -0.270 | 0.125 | -0.270 | -2.154 | 0.036* | 1.874 | 0.534 |
| *R*^2^ | 0.565 | | | | | | |
| Adjusted R^2^ | 0.539 | | | | | | |
| *F* | *F* (3,52)=22.474,*p*=0.000 | | | | | | |
| D-W value | 1.592 | | | | | | |
| Dependent variable = iWUE; * *P* < 0.05 ** *P* < 0.01. | | | | | | | |

| Table S4. Results of multiple linear regression analysis for *S. tzumu*. ((February–September climatic data, *n*=64) | | | | | | | |
| --- | --- | --- | --- | --- | --- | --- | --- |
|  | Unstandardized Coefficients | | Standardized Coefficients | *t* | *P* | Collinearity Diagnostics | |
|  | *B* | Std. Error | *Beta* |  |  | VIF | Tolerance |
| Constant | 0.000 | 0.057 | - | 0.000 | 1.000 | - | - |
| C_a_ | 0.162 | 0.088 | 0.162 | 1.847 | 0.070 | 2.353 | 0.425 |
| VPD | 0.551 | 0.067 | 0.551 | 8.232 | 0.000** | 1.369 | 0.731 |
| T | -0.593 | 0.094 | -0.593 | -6.306 | 0.000** | 2.701 | 0.370 |
| *R*^2^ | 0.803 | | | | | | |
| Adjusted R^2^ | 0.794 | | | | | | |
| *F* | *F* (3,60)=81.769,*p*=0.000 | | | | | | |
| D-W value | 0.796 | | | | | | |
| Dependent variable = iWUE; * *P* < 0.05 ** *P* < 0.01. | | | | | | | |

Table S5. Pearson correlation coefficients between δ^18^O and climatic factors during 2007–2014 for *P. massoniana* (April–October, *n* = 56) and *S. tzumu* (April–September, *n* = 48).

|  | *P. massoniana* | *S. tzumu* |
| --- | --- | --- |
| T | -0.71*** | -0.44** |
| P | -0.37** | -0.27 |
| RH | -0.58*** | -0.38** |
| VPD | -0.12 | -0.05 |

Significance levels: ****P* < 0.001, ***P* < 0.01, *P < 0.05.
